# Therapeutic biomaterials with liver X receptor agonists based on the horizon of material biology to regulate atherosclerotic plaque regression *in situ* for devices surface engineering

**DOI:** 10.1093/rb/rbae089

**Published:** 2024-08-06

**Authors:** Sainan Liu, Jinquan Huang, Jiayan Luo, Qihao Bian, Yajun Weng, Li Li, Junying Chen

**Affiliations:** Key Laboratory of Advanced Technology for Materials of Chinese Education Ministry, School of Materials Science and Engineering, Southwest Jiaotong University, Chengdu 610031, China; Key Laboratory of Advanced Technology for Materials of Chinese Education Ministry, School of Materials Science and Engineering, Southwest Jiaotong University, Chengdu 610031, China; Key Laboratory of Advanced Technology for Materials of Chinese Education Ministry, School of Materials Science and Engineering, Southwest Jiaotong University, Chengdu 610031, China; Key Laboratory of Advanced Technology for Materials of Chinese Education Ministry, School of Materials Science and Engineering, Southwest Jiaotong University, Chengdu 610031, China; Institute of Biomedical Engineering, College of Medicine, Southwest Jiaotong University, Chengdu 610031, China; School of Health Management, West China University, Chengdu 610039, China; Key Laboratory of Advanced Technology for Materials of Chinese Education Ministry, School of Materials Science and Engineering, Southwest Jiaotong University, Chengdu 610031, China

**Keywords:** devices surface engineering, material-biology, pathological microenvironment-regulation, therapeutic biomaterials, atherosclerosis, LXR agonists, macrophage

## Abstract

Percutaneous coronary interventional is the main treatment for coronary atherosclerosis. At present, most studies focus on blood components and smooth muscle cells to achieve anticoagulation or anti-proliferation effects, while the mediated effects of materials on macrophages are also the focus of attention. Macrophage foam cells loaded with elevated cholesterol is a prominent feature of atherosclerotic plaque. Activation of liver X receptor (LXR) to regulate cholesterol efflux and efferocytosis and reduce the number of macrophage foam cells in plaque is feasible for the regression of atherosclerosis. However, cholesterol efflux promotion remains confined to targeted therapies. Herein, LXR agonists (GW3965) were introduced on the surface of the material and delivered *in situ* to atherogenic macrophages to improve drug utilization for anti-atherogenic therapy and plaque regression. LXR agonists act as plaque inhibition mediated by multichannel regulation macrophages, including lipid metabolism (ABCA1, ABCG1 and low-density lipoprotein receptor), macrophage migration (CCR7) and efferocytosis (MerTK). Material loaded with LXR agonists significantly reduced plaque burden in atherosclerotic model rats, most importantly, it did not cause hepatotoxicity and adverse reactions such as restenosis and thrombosis after material implantation. Both *in vivo* and *in vitro* evaluations confirmed its anti-atherosclerotic capability and safety. Overall, multi-functional LXR agonist-loaded materials with pathological microenvironment regulation effect are expected to be promising candidates for anti-atherosclerosis and have potential applications in cardiovascular devices surface engineering.

## Graphical abstract

**Figure:**
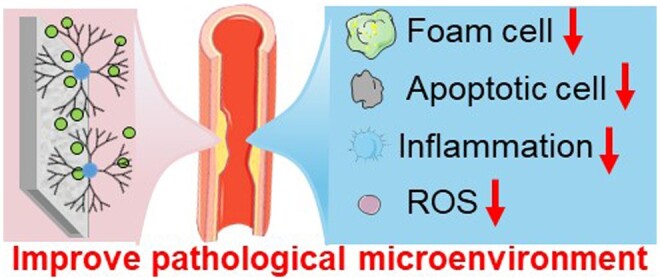

## Introduction

The increasing morbidity and mortality of cardiovascular diseases have become a major public health problem [1]. Clinically, percutaneous coronary intervention (PCI) is the most effective interference means for the treatment of atherosclerosis (AS) apart from drug therapy and surgical treatment. However, the therapeutic efficacy was limited owing to complications such as in-stent thrombosis and in-stent restenosis (ISR) (such as neo-AS) after implantation [2–4]. In view of the problem of insufficient biocompatibility of cardiovascular interventional materials, researchers have screened multiple biomolecules, such as functional molecules, matrix proteins, antibodies and specificity factors [5–8] for device surface engineering, which will regulate the hemocompatibility and cytocompatibility of the surface of the materials respectively or simultaneously, so that the surface has the ability of anticoagulation, anti-restenosis, and intimal repair. However, the microenvironment in the current study, the cells selected for evaluation, and the *in vivo* implantation site are usually in a normal biological state, which is quite different from the cell behavior and characteristics of the atherosclerotic plaque lesion location. In practical applications, the regulatory effects of these biomolecules are influenced by the pathological vascular microenvironment. In addition, combined with the characteristics of atherosclerotic plaques at the device implantation site, there are few studies on the regulation of macrophage behavior and cholesterol molecules. Therefore, this study aims to explore the construction of plaque-adaptive pathological microenvironment on the surface of device materials and use the microenvironment to intervene in the plaque *in situ* to regulate the cellular and molecular biological behaviors and promote plaque regression.

AS is a chronic inflammatory vascular disease characterized by lipid accumulation and inflammatory cell infiltration under the lining of blood vessels, which is a long-term development process caused by dysfunction of macrophages [9]. Macrophages are the most abundant cells in plaques, which can heighten the local inflammation and induce the accumulation of foam cells macrophages-derived in atherosclerotic lesions, leading to the continuous development of AS [10]. More importantly, the risk of rupture was increased due to a high degree of macrophage infiltration in vulnerable plaques [11]. Notably, macrophages are the main balance factor regulating lipid, and the lesion may regress or progress [12] depending on the lipid content and the role of macrophages. The continuous accumulation of free cholesterol in macrophages leads to endoplasmic reticulum stress, which induces an inflammatory response and ultimately leads to apoptosis. The apoptotic bodies are not efficiently cleared, which may lead to atherosclerotic progression [13, 14]. In general, lipid accumulation in macrophages can lead to their dysfunction, affecting the anti-inflammatory, immune and lipid metabolism functions. Liu et al. [15] used multi-enzyme co-expressed nanomedicine to disrupt lipid raft integrity through cholesterol depletion. Additionally, the team [16, 17] alleviated ER stress in the cells through a cholesterol depletion strategy, which metabolically disrupted the immunosuppressive microenvironment and rejuvenated immune cell, thereby restoring immune cell function.

Therefore, reducing cholesterol accumulation in macrophages is expected to be a promising approach to treat and reverse AS. Currently, lipid-lowering therapy using statins has achieved success in clinical [18]. Studies on promoting cholesterol efflux from plaques are also ongoing but are limited to drug-targeting regimens for non-stent therapy which face technical challenges in delivering and targeting plaques. According to research findings [19–21], early thrombosis and acute inflammation occurred after stent implantation, and the inflammation was further aggravated by intima damage and lipid core penetration, suggesting that lipid accumulation and inflammatory cell infiltration play an important role in the development of ISR.

Dendrimer is also attractive for its controllable nano-size structure, a large number of hydrophobic cavities inside, and a high density of cationic functional groups on the surface [22, 23]. Reasonably, the driving forces for the interaction between functional molecules and dendrimer are electrostatic adsorption and covalent reactions between surface groups and molecules, hydrophobic interactions between dendrimer cavities and molecules, and hydrogen bonds between tertiary amines within the dendrimer core and molecules. Dendrimers act as unique carriers to couple a variety of functional molecules, of which are special significance in biomedical applications [24]. Prior work [23] has shown that PAMAM to be a favorable choice as a carrier for good biocompatibility.

Liver X receptors (LXRs) (LXRα and LXRβ), belonging to the nuclear hormone receptor superfamily of ligand-activated transcription factors, have been employed as a therapeutic target for AS due to their versatility in regulating cholesterol homeostasis, inflammation resolution and efferocytosis [25–27]. Once activated, LXRs can regulate the gene expression of ABCA1 and ABCG1, which play an important role in reverse cholesterol transport (RCT). LXRs transport free cholesterol to the liver via ABCA1 and ABCG1, and then promote the excretion of hepatic cholesterol to bile acids and decrease intestinal absorption via ABCG5 and ABCG8 [28, 29]. Thus, LXR plays a guiding significance in inhibiting the formation of foam cells and treating AS. At the macrophage level, Wang et al. [30] found that upon LXR signaling activation, cholesterol efflux to HDL increases, and Sorrentino et al. [31] demonstrated that when lipids accumulated in macrophages, LXR activated could negatively regulate the formation of foam cells, leading to potential anti-atherosclerotic therapy. Further, Zanotti et al. [32] and Wang et al. [33] confirmed *in vivo* the promotion of LXR stimulation on cholesterol RCT from tissues and cells to feces, thereby promoting cholesterol excretion and reducing lipid levels. Inflammation also plays an important role in AS progression. In addition to engendering conducive efficacies on cholesterol efflux, LXR activation also affects atherogenesis by inhibiting the expression of inflammatory genes [34]. Additionally, it was reported that in LXR-deficient mice, downregulated ABCA1 gene expression as well as repressed Mer tyrosine kinase (MerTK) gene expression. Upon activation of LXR, apoptotic cells induced MerTK to reverse defective efferocytosis via the LXR-dependent pathway, thereby eliminating apoptotic cells and resolving inflammation [35]. Accordingly, LXR agonists may exert synergistic anti-atherosclerotic effects by enhancing cholesterol efflux and improving efferocytosis. On the other hand, macrophages can also remove cholesterol from plaques by migrating. The number of foam cells within plaque depends on cell recruitment, *in situ* proliferation, migration and cell death [12]. Research on the progression of AS has focused on understanding the mechanisms by which monocytes accumulate on the vessel wall and designing strategies to prevent their influx into the plaque. However, recent studies have shown that there are other factors that determine macrophage retention and expulsion from plaques, and these factors can lead to a reduction in macrophage number and cholesterol, leading to AS regression [36]. Multiple lines of evidence have shown that LXR is a regulator of CCR7 expression and function, which LXR can have an atheroprotective effect by stimulating the migration of CD68^+^ cells (a marker of macrophages and foam cells) from plaques [37]. At present, massive efforts have been expended to develop agonists of LXRs. Several of these agonists have entered clinical trials, but all were terminated due to adverse events [38]. Two other widely studied LXR agonists, GW3965 and T0901317, are potent LXR agonists that induce *in vitro* and *in vivo* the target genes expression of both LXRα and LXRβ. Despite the positive effects of LXR activation on AS, systemic administration of LXR agonists to an effective dose will cause liver side effects, such as hepatic steatosis and hypertriglyceridemia, which have impeded the progression in clinical [39]. On all accounts, implant materials modified functionally with LXR agonists to target the lesion site *in situ* are necessary for manipulating LXRs and their targets.

**Figure 1.**
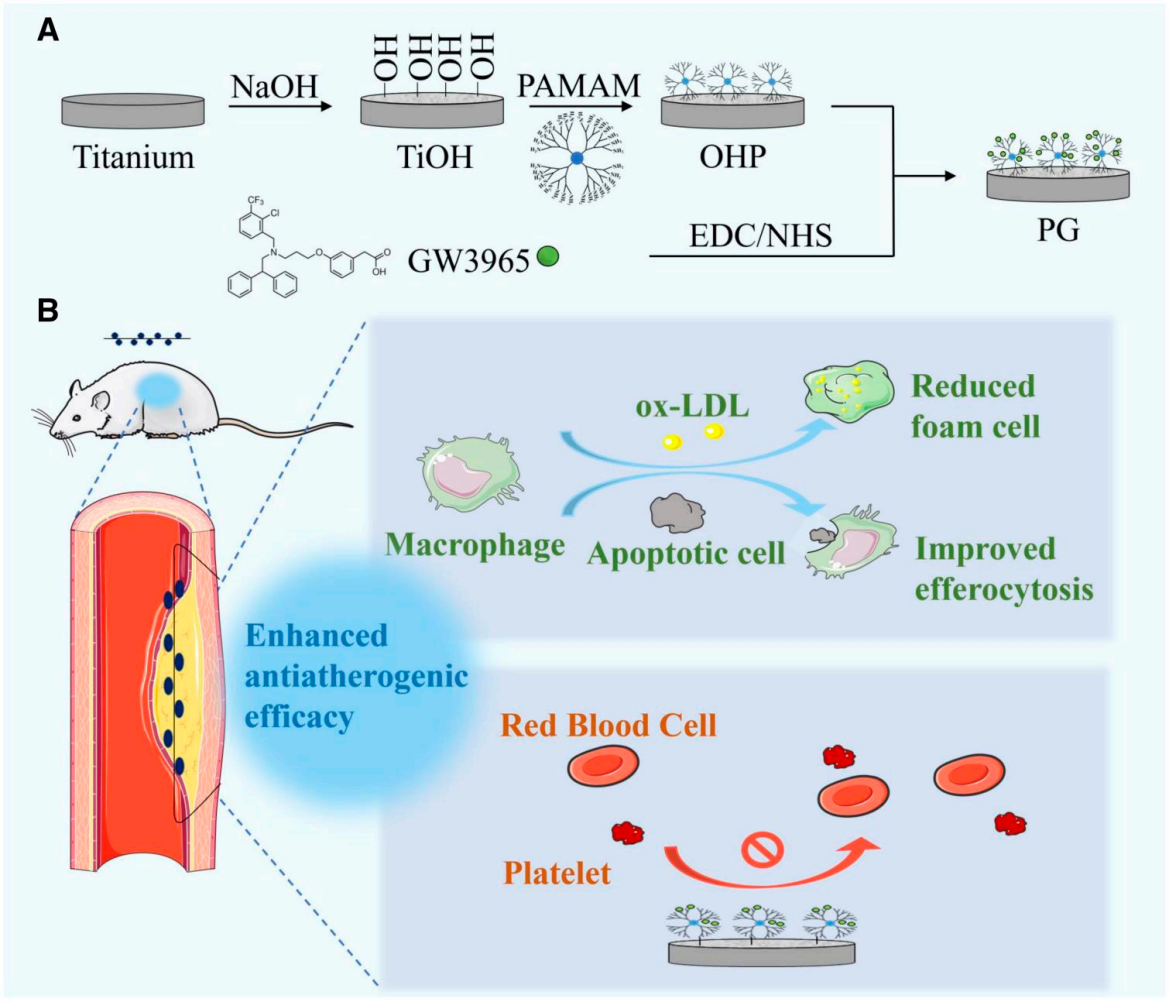
Design of surface modification materials for regulating macrophage function and intervening in the development of atherosclerosis. Schematic illustration of (**A**) the construction of the modified material loaded with liver X receptor agonist and (**B**) the brief treatment diagram for atherosclerosis.

In this work, we used LXR agonist GW3965 to construct a bioactive modified coating on the material surface to regulate lipid content and inhibit atherosclerotic plaque progression. The functional material construction first formed an amine-rich surface with PAMAM immobilized on the surface of alkali-activated titanium via electrostatic interaction. Then, GW3965 was introduced to the surface in both covalent and noncovalent binding, which enhanced the surface fixed amount as well as had a sustained release effect on GW3965. This material can specifically deliver LXR agonist *in situ* to atherosclerotic macrophages, as shown in Figure 1. Predictably, GW3965 acted on macrophages for LXR activation, ultimately enhancing cholesterol effluence and inhibiting inflammatory response, thereby restoring macrophage foam cell migration associated with elevated cholesterol load. In addition, LXR activation could also upregulate the expression of MerTK gene related to efferocytosis to solve the defective clearance of apoptotic cells in AS. Finally, a series of systematic evaluation, including *in vitro* functional test in high-fat cell model simulating pathological environment, *in vivo* safety assessment and *in vivo* anti-AS efficacy evaluation in AS model rats, were conducted to verify its safety and effectiveness.

## Materials and methods

### Materials

G3 PAMAM-NH_2_ was obtained from Weihai CY Dendrimer Technology Co., Ltd (Shandong, China). GW3965 (C_33_H_31_ClF_3_NO_3_) was purchased from Shanghai Yuanye Biotechnology. Oxidized low-density lipoprotein (oxLDL) and 1,1′-dioctadecyl-3,3,3′,3′-tetramethylindo-carbocyanine (Dil)-labeled oxLDL (Dil-oxLDL) were synthesized by Yiyuan Biotechnologies (China). Cell counting kit-8 (CCK-8) was bought from DOJINDO. Total superoxide dismutase (T-SOD) activity assay kit was received from Elabscience (Wuhan, China). Oil red O (ORO) stain kit, total cholesterol (TC) Content Assay Kit and Free Cholestenone (FC) Content Assay Kit were obtained from Solarbio (China). Mouse oxidized low-density lipoprotein (oxLDL) ELISA kit was purchased from Jingmei Biotechnology (Jiangsu, China). Tumor necrosis factor (TNF)-α and interleukin (IL)-10 were obtained from Biolegend. CD86 and CD206 antibodies were provided by Biolegend.

### Preparation of samples

First, pure Ti was treated with NaOH alkaline solution at 80°C to obtain alkali-activated titanium, which is denoted as TiOH. In the ensuing procedures, PAMAM molecules were diluted to 1 mg/ml with RO water. Then, the diluted PAMAM solution was immersed in the TiOH and reacted at room temperature for 12 h to obtain the PAMAM dendrimer-modified amine-rich surface, denoted as OHP. For GW3965 decoration on OHP, the GW3965 molecules were diluted with DMSO. Subsequently, GW3965 molecules were activated by EDC/NHS, reacted at 37°C for 12 h, and covalently grafted to the surface of the amine-rich material, denoted as PG. Based on the different concentrations of GW3965 before the reaction, they were named PG100, PG300 and PG500, respectively.

### Characterization of the material surface components

For detecting the modified layer fixed on Ti, infrared spectrometry was used. The surface of functionalized Ti has a different composition, attenuated total reflection-Fourier transform infrared spectroscopy (ATR-FTIR) was used to measure the surface chemical composition of different samples.

For examining the elemental composition of the modified layer immobilized on Ti, X-ray photoelectron spectra (XPS) were used by an X-ray photoelectron spectrometer. Since the GW3965 molecule contains unique fluorine element, XPS was used to measure the survey scan of different samples and the high-resolution scan of fluorine and to calculate the percent elemental composition of the different sample surfaces.

The effects of PAMAM and GW3965 on the surface morphology of the materials were observed by scanning electron microscopy (SEM) images.

The water contact angle (WCA) of each sample was measured to calculate the changes in hydropathy property before and after modification.

### Characterization of GW3965 quantification and release behavior

The content of GW3965 grafted on the surface was determined with acid orange II and UV-visible spectroscopy. The acid orange II method quantified the amine groups (primary and secondary amines). Briefly, 1 ml of a dye solution dissolved in hydrochloric acid solution (pH 3) was added to the sample surface for 6 h at 37°C. The unconjugated dye was washed off by washing samples with hydrochloric acid solution (pH 3). The bound dye was desorbed in NaOH alkaline solution (pH 12). Finally, the absorbance value was detected at 485 nm using a microplate reader.

Different concentrations of GW3965 were added to the amino-functionalized surface, and the reaction was at 37°C for 12 h, refer to the Preparation of samples section for specific methods. Subsequently, all the solutions after the reaction were extracted, and the absorbance was measured with the UV-visible spectroscopy, and the GW3965 content on the surface was calculated using the difference method.

For evaluating the stability of the GW3965-modified surface, the GW3965 release behavior on the substrate was studied *in vitro*. In brief, the different samples were incubated with 3 ml of PBS solution (pH 7.4) at 37°C and 60 rpm in a gas bath oscillator. At a predetermined time point, the solution in 2 ml of samples was aspirated and 2 ml of fresh-release medium was added to it. The released GW3965 was calculated by UV-Vis spectra.

### Cell culture and foam treatment culture

The mouse mononuclear macrophages RAW 264.7 were cultured in DMEM medium supplemented with 10% fetal bovine serum and with 1% penicillin-streptomycin solution at 37°C in 5% CO_2_ incubator. RAW 264.7 cells were stimulated with 50 μg/ml oxLDL for 24 h to induce foaming [40]. The viability of RAW 264.7 cells and induced foamy macrophages were detected by CCK-8 kit, and live and dead cells were observed with calcein/PI double staining kit, respectively. Additionally, RAW 264.7 cells and induced foamy macrophages were washed and fixed with 4% paraformaldehyde and stained with ORO for 15 min. The formation of induced foamy macrophages was observed via an optical microscope. Further, the cells were lysed and intracellular ORO was extracted to measure the relative concentration at 520 nm by a microplate reader.

### Cell viability assessment

To determine the *in vitro* cytotoxicity mediated by material contacting, the activity of RAW 264.7 cells and induced foamy macrophages was measured via CCK-8 assay. Typically, after the samples were completely sterilized, RAW 264.7 cells and induced foamy macrophages were planted on the surface of all samples placed in a 24-well plate with a density of 5–10 × 10^4^ cells per well. After incubating for 24 h, the samples were immersed in CCK-8 solution and co-cultured for 2 h. The absorbance at 450 nm was measured to calculate the cell viability *in vitro*. In addition, a calcein/PI double staining kit was used to examine both live and dead cells.

### Evaluation of the polarization, anti-inflammatory and antioxidant of induced foamy macrophages

RAW 264.7 cells were treated with oxLDL for 24 h to induce foaming, and induced foamy macrophages were seeded on the surface of samples placed in a 6-well plate with a density of 10^6^ cells per well. After an incubation of 48 h, the cell-free supernatant of each group was collected, the cells were washed with PBS and were collected by centrifugation. To block non-specific proteins, diluted CD16/32 antibody was added to each sample for 15 min. At the end of blocking, the cells were centrifuged and washed with PBS. Following, eFlour 660 (APC) was added and incubated for 15 min for live and dead staining. After that, the mixture was centrifuged and rinsed with PBS. Immediately thereafter, anti-CD86 (PE) was added to each sample group and incubated in darkness at the recommended concentration for 40 min, centrifuged to remove the antibodies, and then resuspended in PBS for flow cytometry (FCM) analysis. Subsequently, another batch of induced foamy macrophages (material-treated) was fixed with 4% paraformaldehyde and treated with a membrane-breaking agent before being detected for CD206 (PE), and the antibodies were incubated successively, in a process similar to that of CD86 [41].

The suspension collected above was centrifuged to remove cell precipitation, and the content of TNF-α and IL-10 was measured by ELISA assay. The specific procedure was carried out according to the instruction of the ELISA kit.

To analyze the antioxidative stress activity of the samples, intracellular superoxide anion was measured by DHE staining and T-SOD kit. Briefly, after co-culture of samples and induced foamy macrophages (refer to the Cell viability assessment section for specific methods), the cells were washed with phosphate buffer (PBS) and stained with DHE (62.5 μM) in serum-free medium for 30 min. Then fluorescence pictures were taken with a fluorescence microscope. Subsequently, the cells were lysed and the intracellular SOD activity was extracted in reference to the instruction of the T-SOD kit.

### Treatment effect of materials on foamy macrophages formation

RAW 264.7 cells were cultured with Dil-oxLDL for 24 h to induce foaming and label intracellular lipids. Subsequently, the cells were washed and were collected by centrifugation to remove excess stain. The induced foamy macrophages were inoculated on the surface and placed on a 24-well plate sample with a density of 7 × 10^4^ cells per well for 48 h. The cells were washed, fixed with 4% paraformaldehyde and stained with DAPI. The cells were washed to remove the stain solution. The intracellular lipid content was observed under a fluorescence microscope and further calculated the mean fluorescence intensity using Image J. Moreover, at the time point of 48 h, the supernatant of another batch of induced foamy macrophages was taken to measure the absorbance at 560 nm by a microplate reader. In addition, the cells were lysed to detect the protein content of each sample with a BCA kit for normalization treatment. In this way, the effect of the materials on promoting cholesterol efflux of cells was characterized.

To quantitatively characterize the content of cholesterol in induced foamy macrophages, for one thing, the content of TC and free cholesterol were measured by TC and FC kits, for another, the content of intracellular lipid was determined by oxLDL ELISA kit. In brief, RAW 264.7 cells were stimulated with oxLDL for 24 h to induce foaming, and induced foamy macrophages were seeded into a 6-well plate sample at a density of 10^6^ cells per well. After 48 h, the cells were washed and collected with centrifugally. The cells were lysed by repeated freeze–thaw followed by ultrasonication, and after that centrifuged and absorbed suspension. The specific procedure was carried out in accordance with the instruction of the ELISA kit. The protein content of each sample was assessed by a BCA kit for normalization treatment.

### Treatment effect of materials on efferocytosis

To evaluate the effect of the material on efferocytosis, the phagocytosis of fluorescent microspheres (Sigma-Aldrich) was observed [35]. Specifically, 50 μg/ml oxLDL-stimulated foam cells were cultured on the sample surface at a density of 7 × 10^4^ cells/well for 24 h. Cells and microspheres (1‰) were incubated in the incubator for 4 h, and the cells were washed with PBS to remove the unbonded microspheres. Then the foam cells were labeled with CellTrace Red CMTPX (YEASEN) and reacted for 30 min in a dark at 37°C. Subsequently, the cells were fixed with 4% paraformaldehyde for 30 min, and the phagocytic microspheres were observed under a fluorescence microscope. At least five images were randomly taken for quantitative analysis of phagocytotic percentage.

### Hemocompatibility assay

To evaluate the blood compatibility of the samples, platelet adhesion and activation test, hemolysis assay, dynamic whole blood experiment and *ex vivo* blood circulation experiment were performed on the materials respectively in New Zealand white rabbits.

The platelet-rich plasma (PRP) and the red blood cells (RBCs) were acquired by centrifuging the fresh blood at 1500 rpm for 15 min. PRP was collected to test platelet adhesion and activation, and RBCs were used for hemolysis experiment. Specifically, 80 μl of PRP was dropped on each sample surface and statically incubated at 37°C. After 45 min, the unfixed platelets were carefully washed with a NaCl solution. Subsequently, Platelets attached to the surface of the samples were immobilized with 2.5% glutaraldehyde, followed by dehydration with gradient alcohol. Finally, the treated samples were imaged by SEM to monitor the morphology and quantitative calculation of the adherent platelets. In the hemolysis assay, a cell suspension containing 2% RBCs was prepared in NaCl solution. Immediately after this step, diluted RBCs interacted with the surface of the material at 37°C for 1 h. RO water and NaCl were used as positive and negative controls, respectively. Then, the reaction solution was centrifuged at 3000 rpm for 5 min and the absorbance of supernatants was recorded at 540 nm to calculate the hemolysis rate.

To analyze the coagulation properties of the materials, whole blood was taken for *in vitro* dynamic whole blood experiment and the blood clotting index (BCI) was calculated [42]. Briefly, fresh non-anticoagulant whole blood was drawn, and blood was dropped on a surface at 100 μl per sample and statically incubated. At the predetermined time (10, 20, 30, 45 and 60 min), each sample was added with 1 ml RO water and still standing for 15 min to lyse RBCs and release free hemoglobin. Then, the supernatant was collected to measure the absorbance at 540 nm, denoted As. Moreover, 100 μl of blood was diluted with 1 ml RO water and kept for 15 min, and the absorbance of the supernatant at 540 nm was recorded as Aw. Finally, BCI was expressed by the ratio of As to Aw to quantify the anticoagulation characteristics of the materials.

To further investigate the blood compatibility between materials and blood in a dynamic environment, an *ex vivo* dynamic circulation experiment was established. Specifically, the male New Zealand white rabbit weighing 1.8–2.2 kg was used, and the sample was placed in a heparin-treated polyvinyl chloride tube, which was connected from the carotid artery on one side of the rabbit to the jugular vein on the other side. Blood flowed out through the separating rabbit carotid artery, and then returned into the jugular vein through the sample tubes to form a dynamic blood circulation with a circulation time of 30 min. Subsequently, the circulating pipes with the samples were rinsed gently with NaCl and fixed with 4% paraformaldehyde solution. Next, the samples were digitally photographed, and the lumen patency diagrams and surface morphologies of each sample were captured. Moreover, after dehydration, SEM characterization was performed.

### Quantitative polymerase chain reaction assay of ABCA1, ABCG1, low-density lipoprotein receptor and CCR7

Gene expression related to the effect of material functional molecules was investigated on the induced foamy macrophage model. First, foamy macrophages were established, that is, oxLDL was applied to RAW 264.7 cells for 24 h to induce foaming, and then foamy macrophages were seeded on the surface of samples placed in a 6-well plate with a density of 10^6^ cells per well. After 48 h, the cells were washed with PBS and collected centrifugally. RAW 264.7 cells in the negative group were treated with DMEM and those in the positive group were pre-incubated with 50 μg/mL oxLDL further treated with DMEM. Subsequently, the cells were lysed using repeated freeze–thaw and ultrasonication and then centrifuged. The supernatant was collected and the expression of LXR intracellular was calculated according to the LXR ELISA kit. Furthermore, the cells were broken up with an RNA extraction solution (Servicebio, G3013) to extract the total mRNA from cells and converted into cDNA using the SweScript All-in-One RT SuperMix for quantitative polymerase chain reaction (qPCR) (One-Step gDNA Remover) kit (Servicebio, G3337). Afterward, quantitative PCR was performed via 2× SYBR Green qPCR Master Mix (None ROX) (Servicebio, G3320). Finally, the gene expression of ABCA1, ABCG1, low-density lipoprotein receptor (LDLR), MerTK and CCR7 was calculated with 2^−ΔΔCT^ method and normalized by GAPDH mRNA, respectively. The primers utilized for GAPDH gene, ABCA1 gene, ABCG1 gene, LDLR gene, MerTK gene and CCR7 gene were as follows. GAPDH-forward 5′ -CCTCGTCCCGTAGACAAAATG- 3′, GAPDH-reverse 5′ -TGAGGTCAATGAAGGGGTCGT- 3′, ABCA1-forward 5′ -AGTCCATCGTGTCTCGCCTGT- 3′, ABCA1-reverse 5′ -GGGATGCTTGATCTGCCGTA- 3′, ABCG1-forward 5′ -TTGTGCTGTTCGCTGCTCTG- 3′, ABCG1-reverse 5′ -GTCACGGGACCCACAAATGT- 3′, LDLR -forward 5′ -GTTCCTGTCCATCTTCTTCCCTA- 3′, LDLR -reverse 5′ -TTCTGGTAGACTGGGTTGTCAAAG- 3′, MerTK-forward 5′ -GCGTGACCATGTGGGAAATAAC-3′, MerTK-reverse 5′ – AAGGGATCAGCACTCCAGCAA-3′, CCR7 -forward 5′ -GAGGCTCAAGACCATGACGGA- 3′, CCR7 -reverse 5′ - ATCCAGGACTTGGCTTCGCT- 3′.

### *In vivo* safety assessment

All animal experiments were approved by the Local Ethical Committee of Southwest Jiaotong University (SWJTU-2103-007, NSFC) and performed in compliance with the Laboratory Animal Administration Rules of China. Sprague–Dawley (SD) male rats (about 220 g) (*n* = 12) were purchased from Chengdu Dossy Experimental Animals Co., Ltd. *In vivo* biocompatibility was inquired via implanting wire into the vessel. Simply, the rats were anesthetized, and the abdominal aorta was separated to guide the pure titanium wire (*D* = 0.1 mm) into the vessel with a 1-ml syringe needle. Then the two ends of the filamentous samples were fixed in the vessel with a suture (9-0) and the abdominal muscle and skin wounds were closed using a suture (2-0) successively, after operation, penicillin was injected. The Ti wires were modified to study experiments *in vivo* by the same operation method, and the groups contained Ti, OHP and PG300 (*n* = 4). After 30 days of feeding in SD rats with implanted samples, the abdominal aorta with the samples were removed and the endovascular lumens were rinsed with NaCl. Then the sample was fixed with a paraformaldehyde solution. Next, the filamentous samples were carefully extracted from the fixed vessels. After paraffin embedding, the vessels were sliced and stained by hematoxylin-eosin (H&E) and immunofluorescence.

### Animal model construction in rats

According to the reports of Fang et al. [43] and Wei et al. [44], the animal model was established as follows. First, all rats (*n* = 10) were fed with a high-fat diet containing 10% egg yolk, 8% lard, 0.2% propyl thiouracil, 0.5% bile salt, 4.8% salt and 76.5% base feed for 7 days. Then the abdominal aorta were exposed to inject liquid nitrogen using a 1-ml syringe needle for frostbitten intima and fed with a high-fat diet for 7 weeks; meanwhile, vitamin D_3_ (50 U/kg, Sanma) was injected on weeks 2, 3 and 4 to accelerate the formation of AS model. At week 5, the abdominal aortic arteries of two rats were randomly sampled for plaque detection. After the time for 7 weeks, 2 ml of blood was taken from the heart of the rats, and placed at room temperature for 2 h, the supernatant was collected after centrifugated at 4°C and 3000 rpm for 15 min to perform four items of blood lipid. Additionally, two aortas of rats were fixed in paraformaldehyde solution to observe the lipid area of the model by gross oil red O staining. The remaining aortas were sectioned after paraffin embedding and the modeling situation was analyzed with H&E. Additionally, after the establishment of the AS model for 7 weeks, the filamentous samples were implanted and fed with high-fat diet for another 4 weeks to observe the curative effect *in vivo*.

### *In vivo* treatment of atherosclerotic plaques

To measure the therapeutic effect of the modified coating, the wire was implanted into the abdominal aorta of the AS rat model and fed with high fat. The rats were evenly separated into Ti, OHP and PG300 groups (*n* = 4). After 4 weeks, the blood vessels were removed for tissue evaluation.

### Statistical analysis

The comparison of multiple groups was analyzed using one-way analysis of variance as well as the difference between the two groups with Student’s *t*-test by Graphpad Prism. All experiments were executed at least three times and shown as mean ± SD (*n* = 3–10). *P* ˂ 0.05 was considered as statistically significant (*P ˂ 0.05, **P ˂ 0.01, ***P ˂ 0.001 and ****P ˂ 0.0001).

## Results

### Preparation and characterization of samples

**Figure 2.**
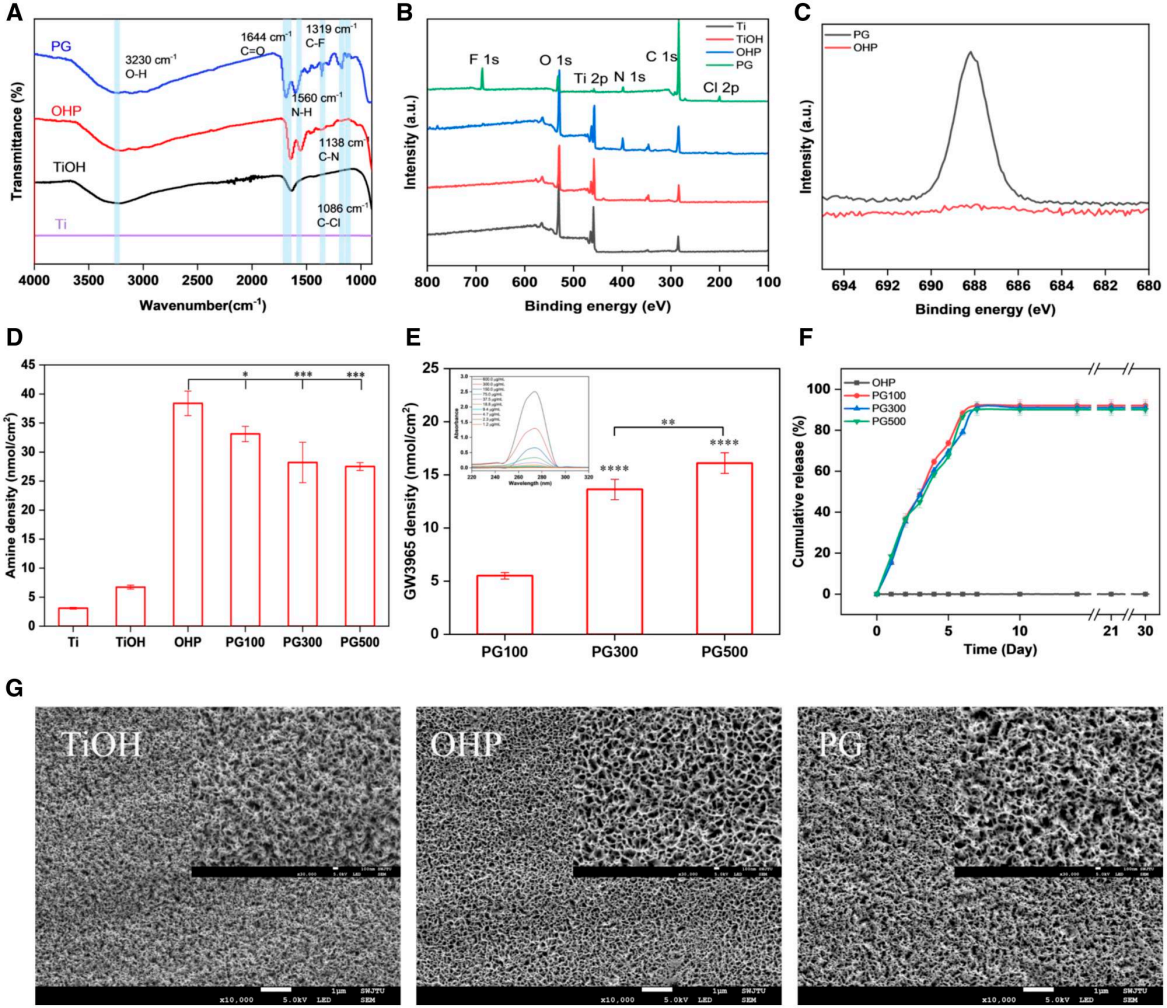
Characterization of materials. (**A**) FTIR spectrum. The survey scan (**B**) and high-resolution scan of fluorine (**C**) of XPS. (**D**) Amine density and GW3965 density (**E**) on the material surface. (**F**) GW3965 cumulative release behavior of material at different time points. (**G**) SEM images of different samples (mean±SD, *N* = 3, *P ˂ 0.05, **P ˂ 0.01, ***P ˂ 0.001 and ****P ˂ 0.0001).

The electronegative surface obtained by the alkaline-heat treatment of Ti can attract PAMAM dendrimers naturally through electrostatic interaction, and the amine-rich surface modified by PAMAM may induce LXR agonist GW3965 to the surface via covalent, electrostatic and hydrophobic interaction. ATR-FTIR was used to detect the group composition on the surface of the modified material. As seen in Figure 2A, there was a broad and strong peak for TiOH at about 3230 cm^−1^, contributed by the O–H vibration. The C=O stretching vibration of OHP and PG appeared at approximately 1640 cm^−1^, and the presence of a peak at 1560 cm^−1^ band confirmed a trace of N–H. The peaks of C–F, C–N and C–Cl of GW3965 appeared at around 1319, 1138 and 1086 cm^−1^, respectively. In conclusion, the FTIR spectra confirmed the fixation of GW3965 on the Ti surface.

As shown in Figure 2B, the XPS full spectrum shows the elemental composition of the specimens. The elemental peaks present in all samples were Ti 2p (456 eV), C 1s (284.8 eV), N 1s (398.4 eV) and O 1s (532 eV). The new peaks for F 1s appeared at 685.7 eV and 199.8 eV for Cl 2p after GW3965 grafting, indicating the existence of fluorine and chlorine contents. Inconsistent with OHP, the peak area of the F element increased significantly for PG (Figure 2C). With the introduction of GW3965, the content of N decreased from 10.33% to 4.25%, while the content of F and Cl increased to 8.82% and 1.89%, respectively (Supplementary Table S1).

As in Figure 2D, acid orange II confirms the amine density. By introducing different concentrations of GW3965, surfaces with different amine densities were generated. Ti/TiOH had the minimum value, and OHP had the maximum density of 39 nmol/cm^2^, the amine density of PG100, PG300 and PG500 gradually decreased, respectively, to 33, 29 and 28 nmol/cm^2^. After 30 days of release, the surface amine density was still detectable with the same trend (Supplementary Figure S1). Besides, the content of GW3965 in solution was detected by UV at 271nm wavelength, and the surface grafting amount was calculated by differential method. As could be seen from Figure 2E, the density of GW3965 increased successively, which were 6, 14 and 16 nmol/cm^2^, respectively. The drug release behavior of the modified materials in PBS was measured and recorded in Figure 2F. The release patterns of PG100, PG300 and PG500 were similar at different concentrations. PG100, PG300 and PG500 were released rapidly within 2 days, reaching more than 35%. The release rate of each sample decreased slightly from 3 to 7 days, and leveled off after 10 days, with a cumulative release of more than 90%. In conclusion, the GW3965 modified material has a certain stability and has the effect of sustained release of drugs, which can improve the utilization rate of drugs.

In addition, the surface morphology of the materials was observed by SEM images, as shown in Figure 2G. After alkali activation treatment, the surface of titanium was covered with a layered porous structure, which made the surface have high hydroxyl sites and negative charge while increasing the surface roughness. After the introduction of the PAMAM-modified layer, the random dense micropores became more visible and slightly consistent, probably due to the reduced surface roughness caused by the introduction of PAMAM. After fixing GW3965, the surface then becomes dense, resulting in the surface pore size of GW3965 modified material between TiOH and OHP. According to the results of WCA (Supplementary Figure S2), the WCA decreased from 63° to 17° after alkali activation treatment, while the WCA further decreased to 11° after PAMAM modification.

### Effects of materials on MA and OxLDL-stimulated MA

**Figure 3.**
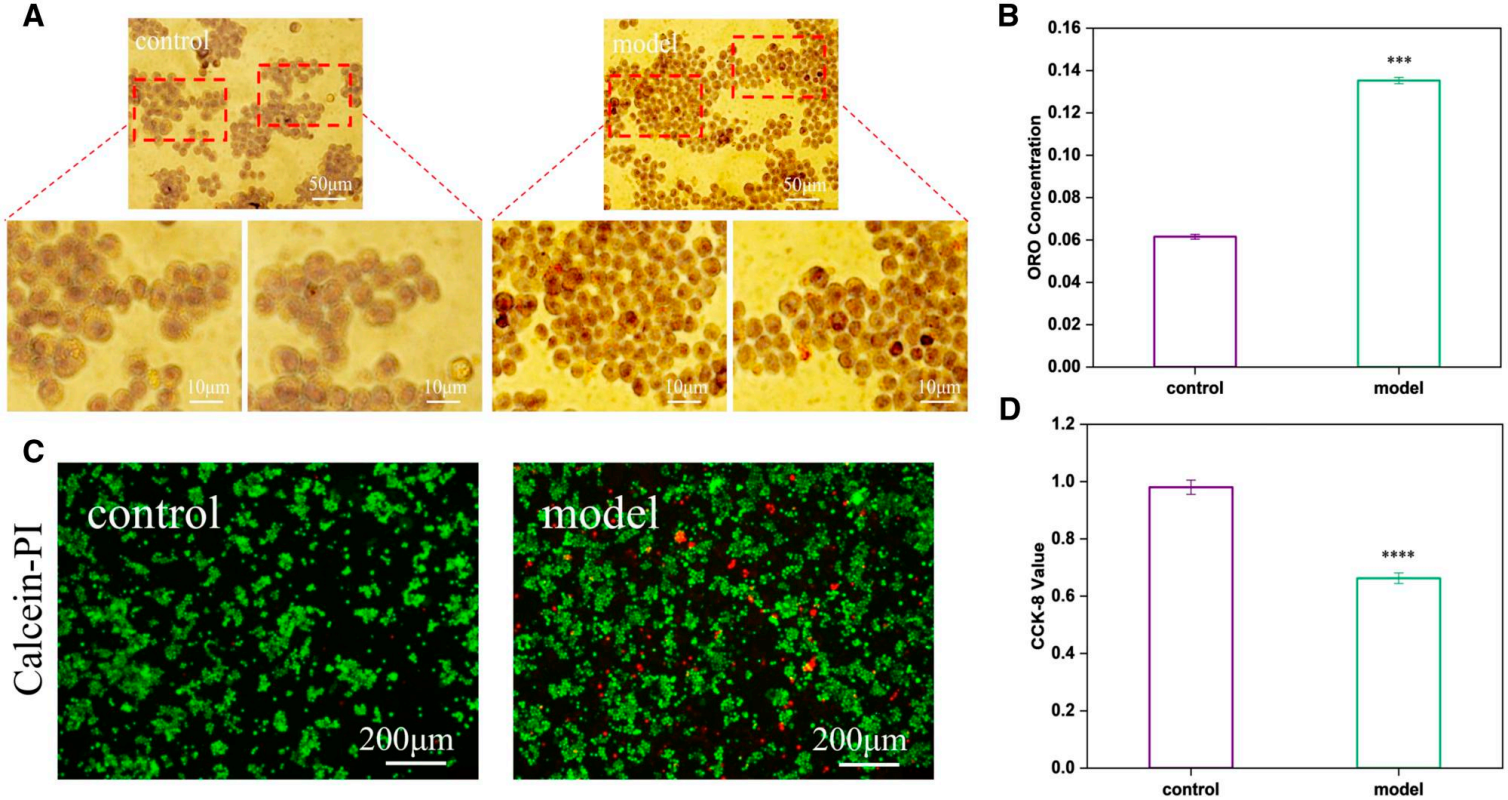
*In vitro* establishment of foam cell model. (**A**) Intracellular lipid staining under bright field and (**B**) intracellular dye concentration. (**C**) Fluorescent images of Calcein-PI staining of live and dead cells and (**D**) cell viability. (mean±SD, *N* = 3, ***P ˂ 0.001, ****P ˂ 0.0001).

Macrophages are the earliest cellular components in atherosclerotic plaques and markers of plaque formation and inflammation. Stimulated by persistent inflammation, macrophages participate in lipid metabolism, secrete inflammatory factors and form foam-like cells that in the worst case develop into apoptotic or necrotic cell [45]. Hence, the influence of the sample on the characteristics of macrophages is the main factor to be investigated. Primarily, macrophages were stimulated with oxLDL to simulate the formation of foamy macrophages *in vitro*, to explore the influence via co-cultured with samples. The foamy macrophages were stained with oil red O and the staining was observed under a light microscope, it was obvious that the lipids in the foamy macrophages were dyed red (Figure 3A). Furthermore, the absorbance of the intracellular stain was measured, and the relative content of the stain in the foamy macrophages was higher than that in the macrophages (Figure 3B). In addition, live and dead staining and activity determination were performed, and the model foamy macrophages had obvious dead cells and their activity was reduced by 40% (Figure 3C and D). With the increase of oxLDL concentration, the activity of macrophages decreased, and the lipid content increased, as well as intracellular reactive oxygen species (ROS) (Supplementary Figure S3), indicating that the *in vitro* cell model was successfully constructed.

**Figure 4.**
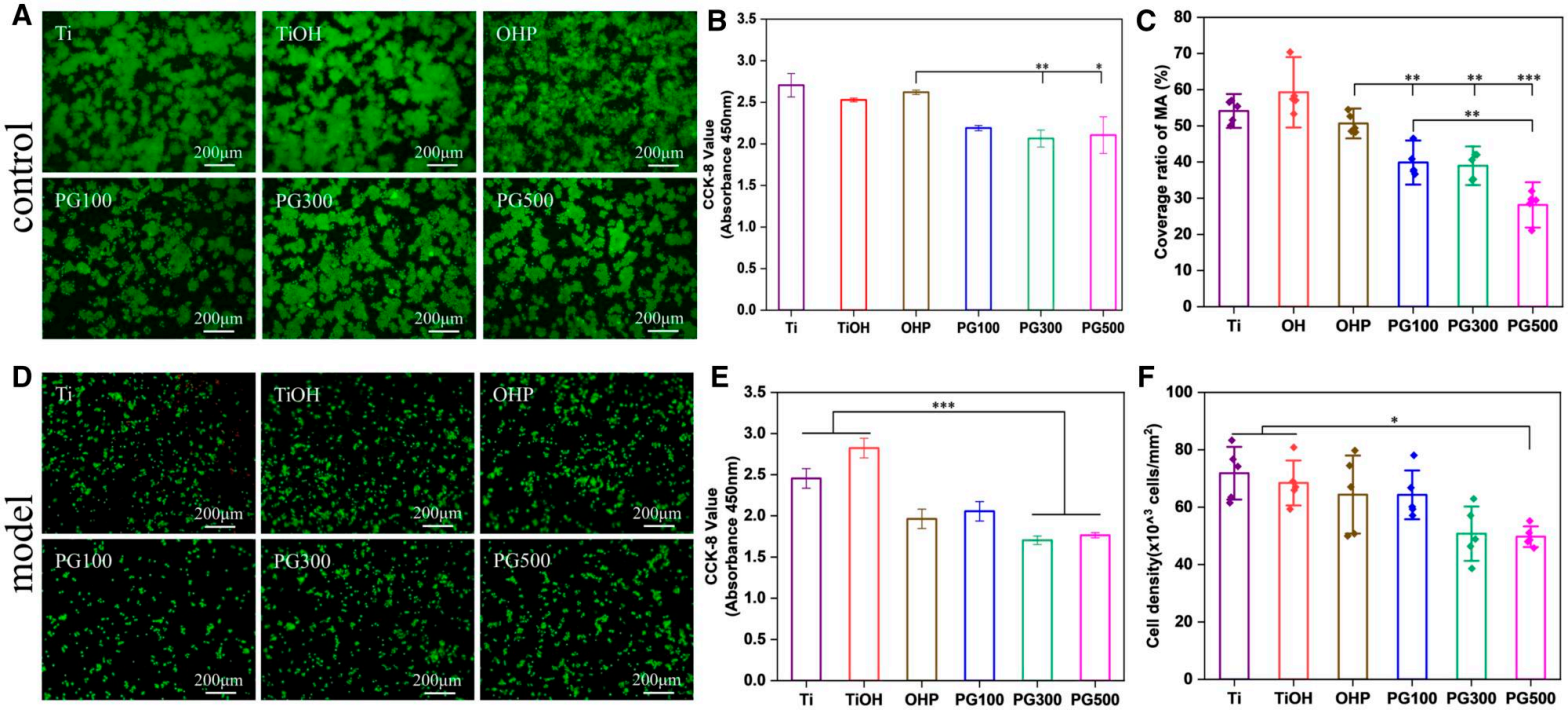
Cytocompatibility of materials. (**A**, **D**) Fluorescence images of MA and foam cells. (**B**, **E**) CCK-8 results and (**C**, **F**) number of cells adhesion of two types of cells. (mean±SD, *N* ≥ 3, *P ˂ 0.05, **P ˂ 0.01, ***P ˂ 0.001).

There are differences between the foam cell model and macrophages at the cellular level, such as phenotype, as well as at the molecular level, including inflammation, lipid and ROS levels. Therefore, we explored the effects of each sample on various aspects of foam cell function to provide a basis for the study of the pathological environment of AS *in vivo*. Following, MA and the model cells were inoculated on the surface of the sample to observe the cells' activity and adhesion. The live and dead cells were located with Calcein-PI dye, in which the Calcein-labeled live cells presented green fluorescence, whereas the PI-labeled dead cells showed red fluorescence, meanwhile, the cell activity was measured. The results of MA in Figure 4A showed that hardly any dead cells were present in all groups but different live cell coverages. Figure 4B showed a decrease in the activity of the cells. Similarly, the cell adhered on the surface of GW3965 was significantly lower than that of other groups, and the cell coverage of OHP was 1.2 times, 1.3 times and 1.9 times that of PG100, PG300 and PG500, respectively (Figure 4C). The results of the model cells are shown in Figure 4D–F. GW3965 could effectively reduce the activity and inhibit the adhesion of foamy macrophages *in vitro*, which there was indiscriminate among different concentrations. In short, GW3965 can effectively inhibit the proliferation of inflammatory macrophages *in vitro*, and there is no significant difference between different concentrations.

### Evaluation of *in vitro* anti-inflammatory, antioxidant, anti-foam cell formation and efferocytosis

**Figure 5.**
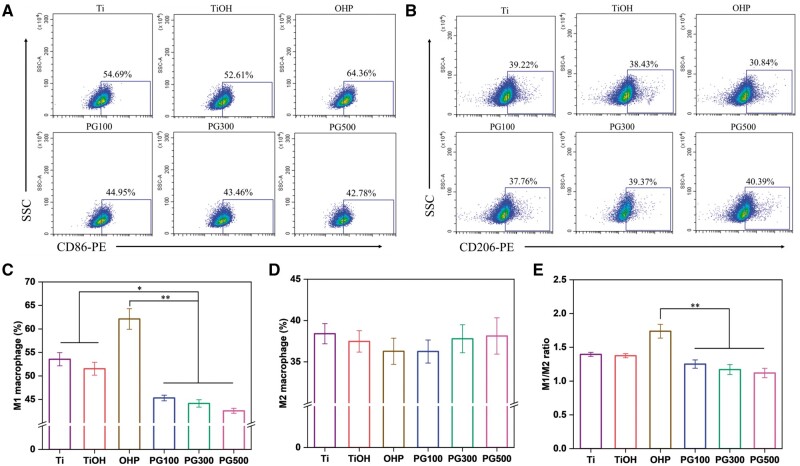
Effect of different materials on macrophage polarization *in vitro*. (**A**, **B**) FCM analysis of the proportion of (**A**) M1 macrophages (labeled with CD86+) and (**B**) M2 macrophages (labeled with CD206+). Percentages of (**C**) CD86+ and (**D**) CD206+ cells. (**E**) The ratio of M1 to M2 (mean±SD, *N* = 3, *P ˂ 0.05, **P ˂ 0.01).

**Figure 6.**
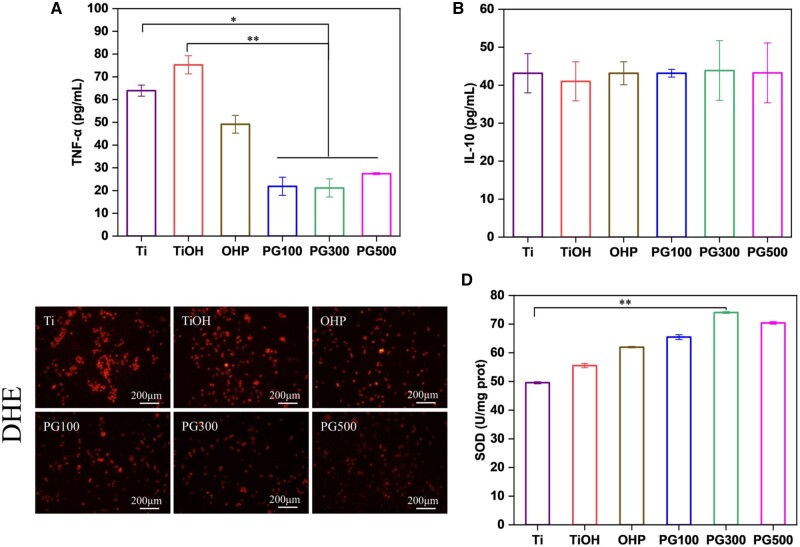
Characterization of intracellular inflammatory factors and ROS level after incubation with different materials. Cytokines content of (**A**) TNF-α and (**B**) IL-10 tested by ELISA. (**C**) Fluorescence images of ROS labeled by DHE and (**D**) activity of SOD (mean±SD, *N* = 3, *P ˂ 0.05, **P ˂ 0.01).

M1 macrophages secrete proinflammatory factors to accelerate the development of inflammation, whereas anti-inflammatory factors are secreted by M2 macrophages to effectively resist inflammation, so the phenotype of macrophages plays an important role in AS. In this study, oxLDL exposed macrophages to a stimulating environment, thereby transforming them into M1 phenotype. In other words, M1 macrophages were taken as the object to investigate the effect of samples on the polarization via FCM. Consequently, GW3965 significantly reduced the number of M1 compared to OHP. Notably, PG300 effectively inhibited M1 macrophages at 27%, PG300 at 30% and PG500 at 31% (Figure 5A and C). In contrast, GW3965 slightly increased the number of M2 macrophages, and there was no significant difference in the positive rate of M2 macrophages among different samples (Figure 5B, D and E). To further analyze the polarization of macrophages, ELISA kit was employed to detect the secretion of TNF-α by M1 and IL-10 by M2 macrophages. As shown in Figure 6A and B, GW3965 could significantly downregulate the secretion of TNF-α; however, it has not obviously influence on IL-10. It is worth noting that the effective inhibition rate of GW3965 on TNF-α was more than 50% of OHP. The results were consistent with the FCM. In conclusion, GW3965 decreases the number of M1 macrophages and downregulates the secretion of inflammatory factor TNF-α, thus achieving the effect of inhibiting inflammation.

Since oxLDL is renowned for its oxidation feature, the antioxidant activity of the sample was tested. Intracellular ROS can be characterized qualitatively by DHE probes labeling, displaying red fluorescence. Further, SOD activity was determined to quantitatively characterize ROS. Compared to control groups, the SOD activity of PG100, PG300 and PG500 was increased by approximately more than 1.1, 1.2 and 1.3 times, respectively, which the antioxidant activity of PG300 was more obvious (Figure 6C and D).

**Figure 7.**
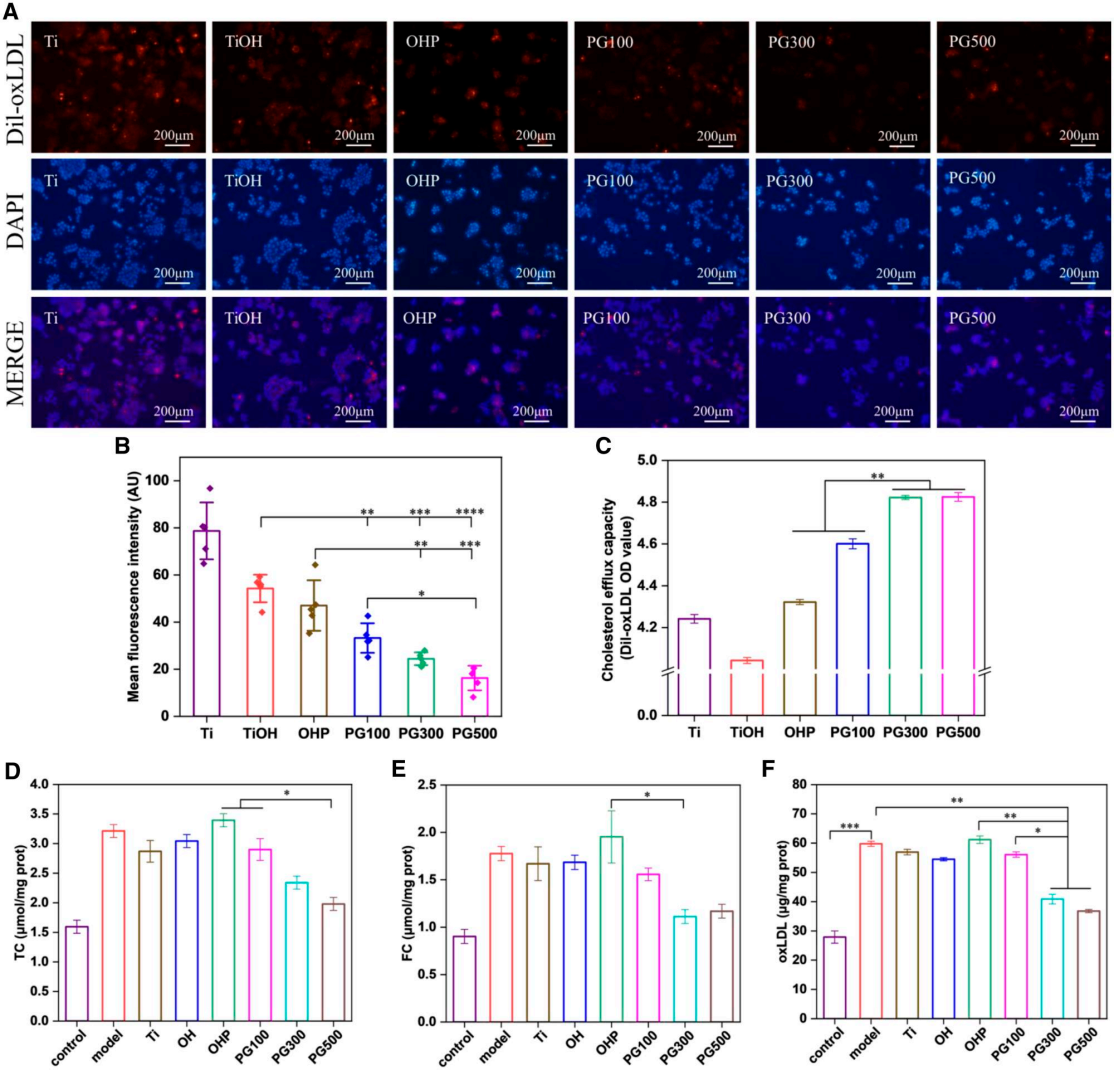
Effects of different materials on intracellular lipids. (**A**) Fluorescence images of dil-oxLDL and (**B**) comparison of mean fluorescence intensities. (**C**) Presentation of intracellular cholesterol efflux by different samples. (**D**–**F**) Intracellular content of TC, FC and oxLDL in macrophages, foam cells and cells treated with different samples (mean±SD, *N* ≥ 3, *P ˂ 0.05, **P ˂ 0.01, ***P ˂ 0.001 and ****P ˂ 0.0001).

Macrophages are not only involved in inflammation of AS but also closely related to lipid metabolism. When lipid uptake and efflux of macrophages are out of balance, lipid accumulation in macrophages will result in the formation of foamy macrophages, which generates a series of serious consequences such as the weakening of the migration ability of macrophages and the enlargement of plaque. GW3965 is well known as an LXR agonist that promotes cholesterol efflux, however, whether GW3965-modified materials have this effect has not been cleared. Therefore, the changes in lipid content in macrophages treated with the material were investigated. Initially, intracellular lipids were labeled with Dil-oxLDL and showed red fluorescence. In Figure 7A and B, the sample loaded with GW3965 reduced the positive staining area of lipid. The mean fluorescence intensity of lipid decreased in the following order: Ti, TiOH, OHP, PG100, PG300 and PG500. In addition, the mean fluorescence intensity of Ti was 2, 3 and 5 folds that of PG100, PG300 and PG500, respectively, indicating GW3965 possessed the potential to inhibit foamy macrophage formation. On the other hand, the cholesterol efflux capacity of samples was assessed by measuring the content of Dil-oxLDL in the supernatant as shown in Figure 7C. GW3965 showed enhanced cholesterol efflux, among which PG300 and PG500 showed the most significant and comparable lipid clearance. Further, lipid content was assessed by intracellular TC, free cholesterol (FC), and oxidized low-density lipoprotein via the kit. Figure 7D–F showed that the lipid content of model cells is more than twice that of untreated macrophages, and after Ti, TiOH and OHP treatment, there is no significant difference in lipid content. By contrast, lipid content is significantly reduced with GW3965 treatment, and the foamy macrophage formation is most significantly inhibited in PG300 and PG500. These results together demonstrated the effect of GW3965 in promoting cholesterol efflux, reducing lipid accumulation, and effectively inhibiting the formation of foamy macrophages.

**Figure 8.**
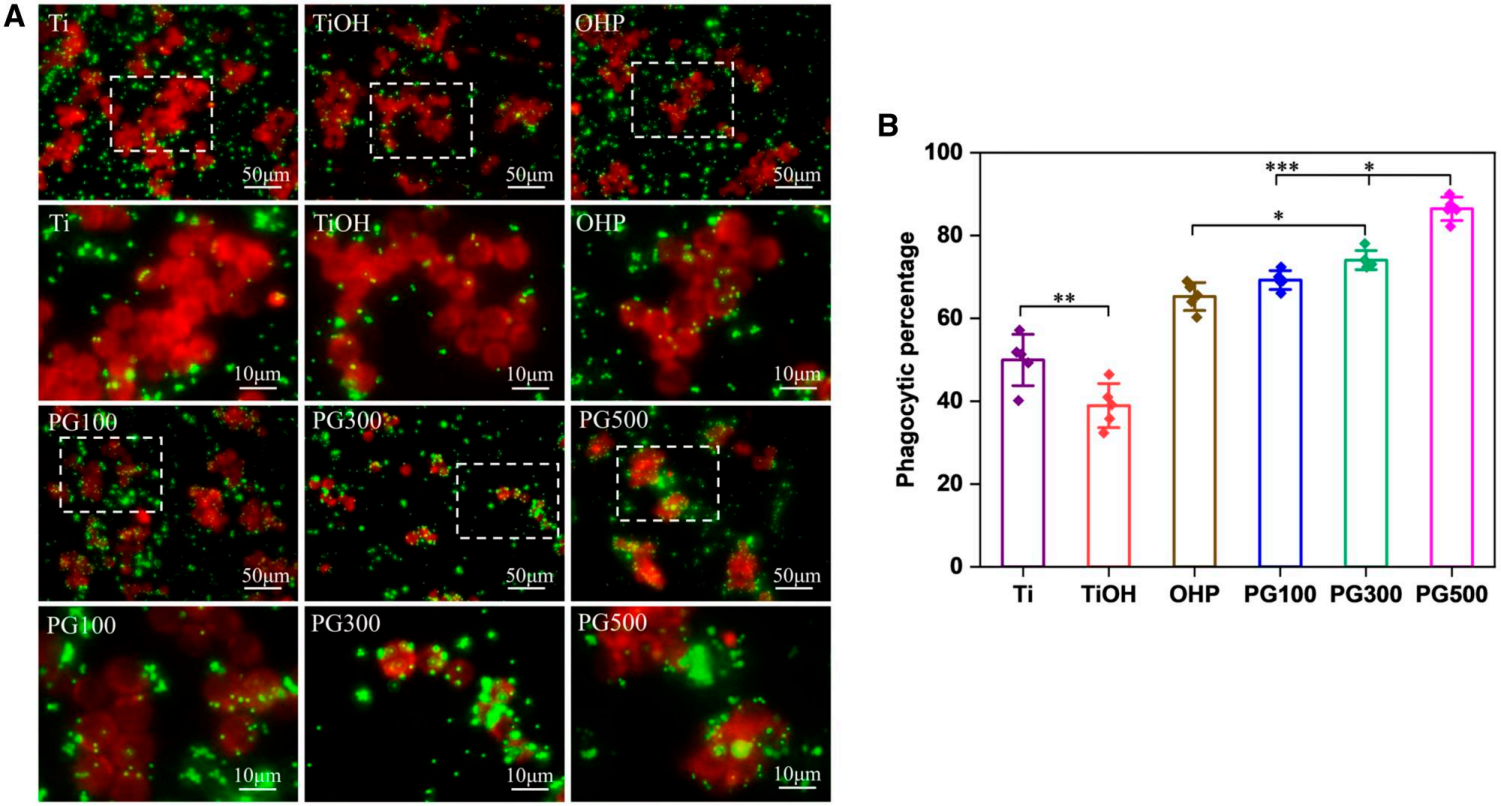
Effects of different materials on phagocytosis. (**A**) Fluorescence images of microspheres incubated with CellTracker Red CMTPX-labeled foam cells on the sample. (**B**) Quantification of phagocytic function of foam cells treated with different samples (mean±SD, *N* ≥ 3, *P ˂ 0.05, **P ˂ 0.01, ***P ˂ 0.001).

In AS, macrophages exhibit defective efferocytosis, which exacerbates the inflammatory and necrotic cores. Fluorescent microspheres were used to observe the phagocytosis of foam cells after sample treatment. Figure 8A showed the fluorescence images of phagocytosis of microspheres, and it could be seen that the number of cells in Ti and TiOH groups exhibiting microsphere phagocytosis was relatively small, and the distribution of microspheres was dispersed. In contrast, the cells treated with the GW3965-modified material showed stronger phagocytosis, and more microspheres accumulated around the cytoplasm and membrane. According to the statistical results in Figure 8B, compared to the OHP group, the endocytosis capacity of PG500-treated foam cells to microspheres was increased by approximately 21%.

### Hemocompatibility properties

**Figure 9.**
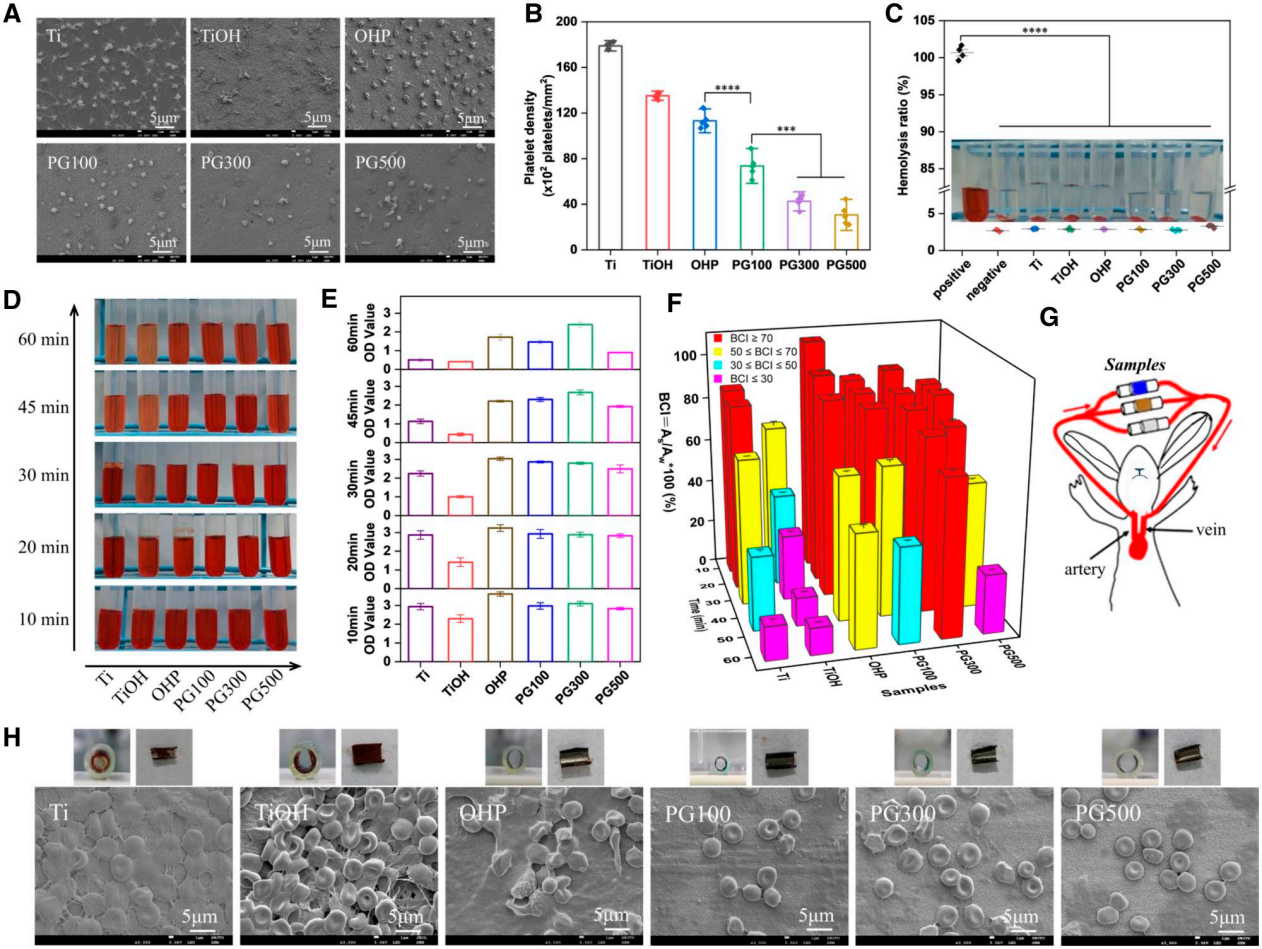
Hemocompatibility of materials. (**A**) SEM images and (**B**) number of platelet adhesion on samples. (**C**) Hemolysis ratio of different samples. (**D**) Digital pictures and (**E**) absorbance values of the *in vitro* dynamic coagulation assay for different samples. (**F**) BCI statistical graph. (**G**) Illustration of *ex vivo* model. (**H**) Cross-sectional images of catheters with samples and photographs of the sample after unfolding and SEM images of material surfaces after dynamic blood circulation for 30 min (mean±SD, *N* ≥ 3, ***P ˂ 0.001, ****P ˂ 0.0001).

Implant materials that are directly in contact with blood will interact with blood components, leading to thrombosis and hemolysis. Therefore, blood compatibility are crucial problem to reflect the interaction of biomaterial and blood. Platelet adhesion, activation and aggregation occur on the surface, and progress toward the process of thrombosis, which will eventually affect the blood flow. In this part of the study, platelet adhesion and activation were determined. The results showed that there were massive adhered platelets on both Ti and TiOH. Compared with the extended pseudopodia and aggregation of platelets on Ti, TiOH showed a completely spread state, indicating that the material could induce the activation of platelets after alkali activation. The number of platelet adhesion in the PAMAM transition layer decreased by about 37% compared to Ti control, and most platelets presented normally round. For the experimental group introduced with GW3965, the number of platelet adhesion was further reduced. In contrast to the OHP control group, GW3965 inhibited platelet adhesion by approximately 35%, 60% and 73%, respectively, from low to high concentrations (Figure 9A and B), indicating a higher anticoagulant activity in a concentration-dependent manner. Besides, we performed a hemolysis test, which was an important experiment to evaluate blood compatibility. It turned out that the supernatants of all samples except the positive control were pellucid (Figure 9C). The hemolysis ratio of all samples was below 5%, which met the safety standard.

*In vitro* whole blood dynamic coagulation test is often measured to assess blood clotting properties. When materials encounter blood, a clotting reaction occurs, RBCs not trapped in the clots dissolve in water and rupture, releasing hemoglobin. Therefore, the degree of clotting was distinguished by the color of the collected solutions, and the absorbance values were also recorded, as shown in Figure 9D and E. The higher the absorbance value, the freer RBCs dissolved in the water, thus the fewer RBCs coagulated on the sample, indicating that the anticoagulant effect of the material is better. Within 30 min, all the samples, except TiOH, had good anticoagulation. After 30 min, the absorbance of the sample decreased sharply, and the absorbance of TiOH was the lowest, indicating that the most severe clotting occurred on its surface, followed by Ti. Among them, PG300 had hardly a change in absorbance, decreasing by only 0.6. Corresponding to this, macro-graphs of the liquid collected by adding RO water after the formation of clots on the surface showed a consistent trend. Lighter red indicated less effective anticoagulation, and among these, PG300 showed the least color change. In addition, a high value of the BCI indicates that the dissolved hemoglobin is more, reflecting that the material requires a long coagulation time. Figure 9F shows that with the extension of time, BCI value decreased, and BCI value of PG300 was about 71%, which was 5 times that of Ti control and 1.6 times that of the OHP control. In the experimental group with different concentrations, PG300 also revealed excellent anticoagulation performance.

To further study the hemocompatibility under a flowing blood environment, an *ex vivo* experiment was conducted as clarified in Figure 9G, in which samples were curled on the inner wall of the catheter. Significant occlusion occurred in the Ti and TiOH, while no occlusive thrombosis was observed in the lumen of the other samples (Figure 9H). SEM images revealed that dense thrombus was formed on the surface of Ti and TiOH, characterized by a fibrin network with substantially activated platelets and captured RBCs. Nevertheless, the fibrin network was not apparent on the surface of GW3965, and platelet attachment and RBC capture were significantly reduced. Taken together, PG300 consistently exerted the efficient antithrombosis ability within 60 min.

**Figure 10.**
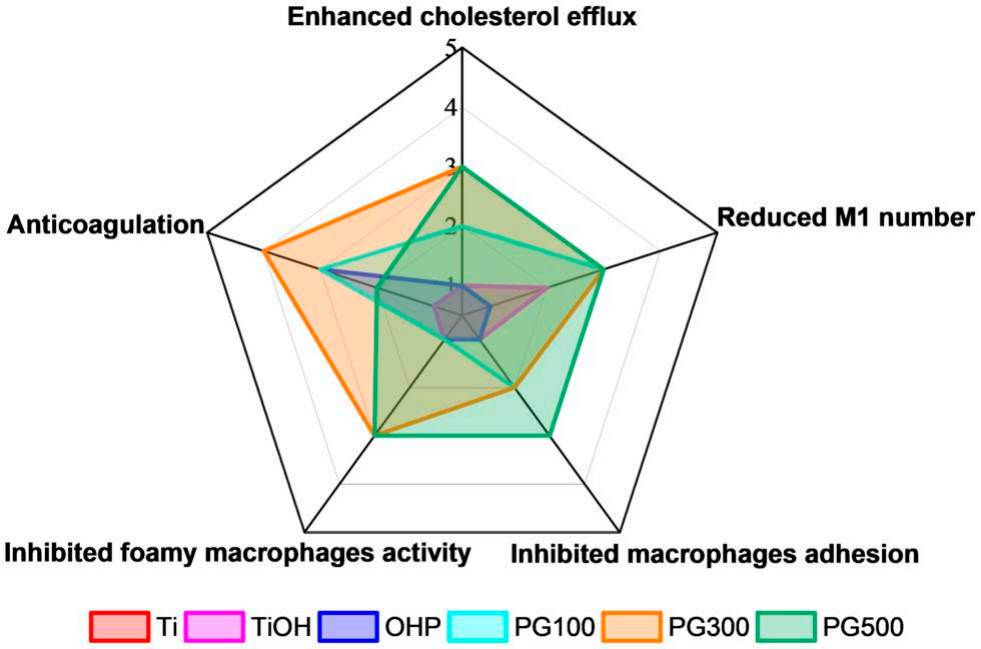
Comprehensive evaluation of *in vitro* experiments for each sample.

Combined with the above *in vitro* effects of various samples, including inhibiting the activity and adhesion of macrophages and foam cells, reducing the number of inflammatory macrophages, anticoagulation and promoting cholesterol efflux, it turned out that GW3965 modified materials had corresponding benefits as the drug itself, among which PG300 has the best performance (Figure 10). Therefore, samples with PG300 concentration were selected as the experimental group, and Ti and OHP were used as the control group to verify the *in vivo* treatment and explore the mechanism.

### mRNA expression of materials treated foamy macrophages

**Figure 11.**
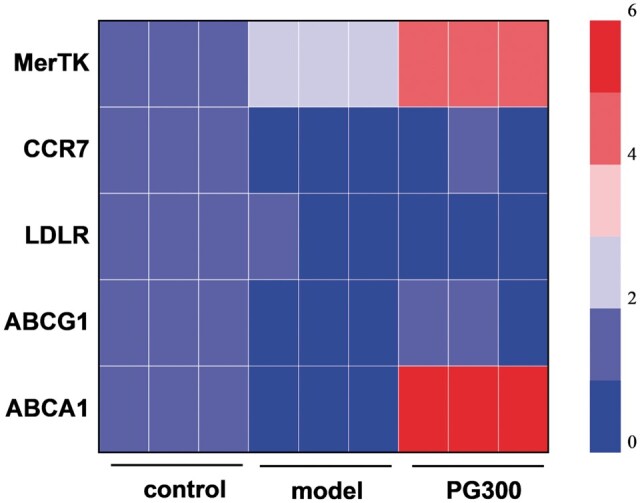
Changes of mRNA expression of ABCA1, ABCG1, LDLR, MerTK, CCR7 in macrophages after PG300 treatment (mean±SD, *N* = 3).

As we all know, GW3965 is an agonist for LXR, which plays a role in the whole process of RCT. The target genes regulated by LXR include the ABC family. In macrophage experiments, it was found that it upregulated the expression of ABCA1 and ABCG1, which transported cholesterol to HDL for metabolism and reduced the formation of foamy macrophages. In this section, we examined the levels of various target genes in model foamy macrophages to validate the signaling pathways that promote cholesterol efflux *in vitro* (Figure 11). qPCR showed that the target genes ABCA1 of foamy macrophages with PG300 treatment were about five folds that of model cells, indicating that GW3965 significantly upregulated the expression of ABCA1 and slightly upregulated the expression of ABCG1. Cholesterol enters cells through the LDLR, and Figure 11 shows that PG300 downregulated the expression of the LDLR gene, thereby reducing cholesterol uptake. The weakened migration ability of macrophages in atherosclerotic plaques leads to their accumulation and intensification of inflammation. Therefore, enhancing the migration of macrophages can effectively inhibit inflammation, and CCR7 facilitates the migration of macrophages, so the gene expression level of CCR7 was detected. The results indicated that GW3965 can be observed to reverse oxLDL-induced downregulation of CCR7 and enhance its activity. Finally, the upregulation of MerTK gene in PG300 which mediated efferocytosis, implied that the material can restore the defective efferocytosis in plaques. The aforementioned genes were all the downstream signaling pathways of LXR; therefore, the expression of intracellular LXR was also detected as the most critical. The results of Supplementary Figure S4 showed that PG300 effectively upregulated the expression of LXR. In summary, the material loading GW3965 could activate LXR, which could reduce the formation of foam cells by reducing the uptake of lipoprotein and increasing the efflux of cholesterol, reduce the accumulation of inflammatory macrophages by accelerating the migration, and reduce the necrotic core by enhancing the clearance of apoptotic cells, thus achieving anti-atherosclerotic effects.

### Evaluation of materials implantation therapy in vivo for AS

**Figure 12.**
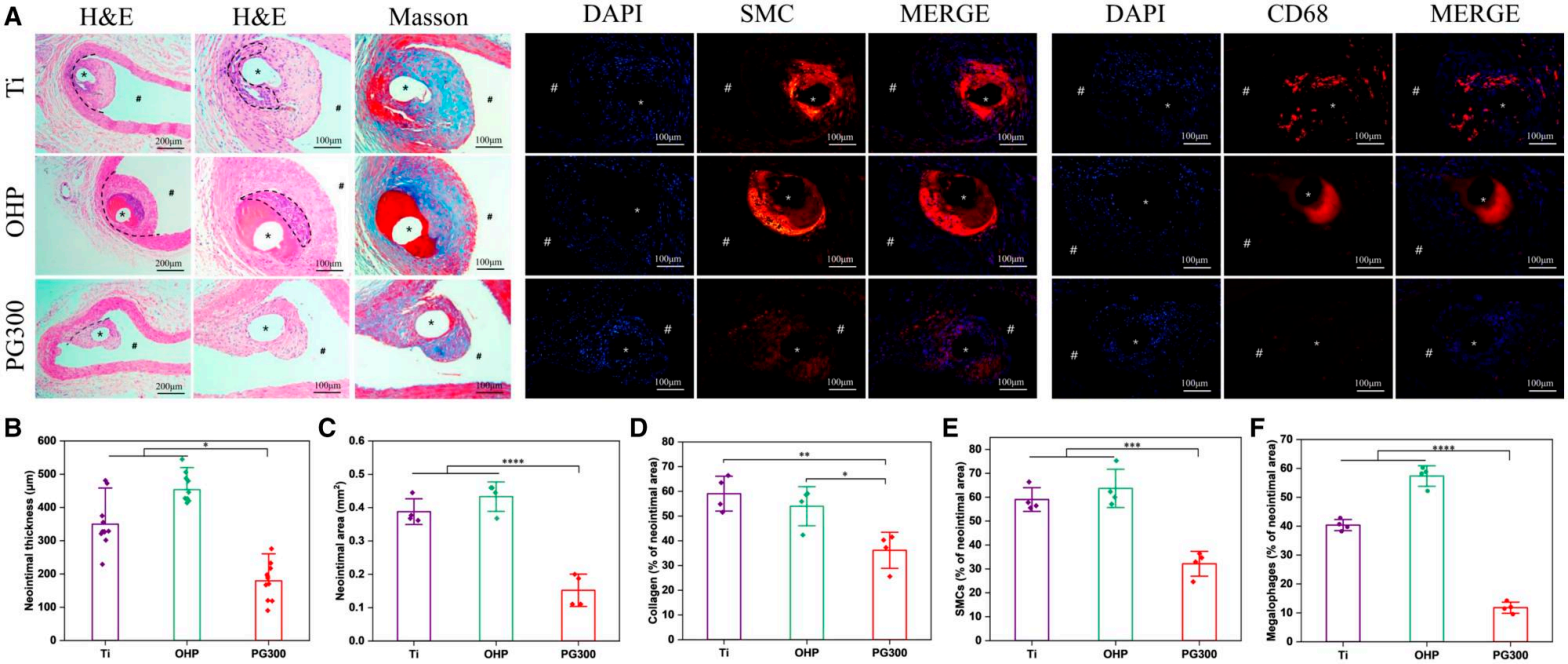
*In vivo* safety assessment. (**A**) H&E and Masson staining and immunofluorescence staining images of SMCs (labeled by α-SMA) and MA (labeled by CD68) of the sample after implantation in the rat abdominal aorta for 30 days (*: sample implantation site, #: intravascular lumen). Statistics of (**B**) neointimal thickness and (**C**) neointimal area at implant site. Percentage of (**D**) collagen, (**E**) SMCs and (**F**) macrophage in neointima (mean±SD, *N* ≥ 3, *P ˂ 0.05, **P ˂ 0.01, ***P ˂ 0.001, ****P ˂ 0.0001).

The implantation of samples is a process of introducing foreign bodies into the body. The materials will interact with blood and vascular wall tissues, and the neonatal tissues will gradually enclose the implanted samples over time. Here, we evaluated material and tissue interactions *in vivo* by implanting titanium wire, OHP which was PAMAM-modified material, and PG300 specimens introduced LXR agonist into the abdominal aorta of SD rats, respectively. After 30 days of implantation, samples from each group were coated with neointima. The results of H&E staining in Figure 12A showed that there were extensive inflammatory cells gathered in the innermost sites of neointima in Ti and OHP groups (dotted line circled area), while there were no obvious inflammatory cells in PG300, indicating lighter inflammation reaction. Compared to Ti and OHP, neointimal thickness was reduced by about 49% and 60% in PG300, respectively (Figure 12B), and neointimal area was both reduced by more than 60% (Figure 12C). In addition, PG300 had less smooth muscle cells and macrophages, resulting in less neointimal hyperplasia in the sample. Proliferated smooth muscle cells and aggregated inflammation within the neointima of Ti and OHP led to the production of increased collagen fibers (Figure 12D–F). To further analyze the level of inflammation in the tissue, macrophage phenotypes were specifically stained. Proinflammatory macrophages presented positive expression of iNOS and were stained with green fluorescence, which enhanced the inflammatory response of surrounding tissues by secreting inflammatory factors, while anti-inflammatory macrophages specifically expressed CD206 and were stained with red fluorescence, which inhibited and eliminated the inflammation of tissues by secreting anti-inflammatory factors. As shown in Supplementary Figure S5A, the expression of CD206 was weak around sample implantation, and iNOS was not detected obviously as positive, which might be due to that the material did not introduce a serious inflammatory response. It partially might also cause acute inflammation, which is subsided by the body's self-regulation after long-term implantation. Through statistical calculation (Supplementary Figure S5B), the M2/M1 ratio around PG300 samples was higher, indicating that M2-positive cells expressed more, which was conducive to the regression of inflammation. The above results confirmed that the GW3965-modified material enhanced the histocompatibility and effectively inhibited the occurrence of restenosis.

**Figure 13.**
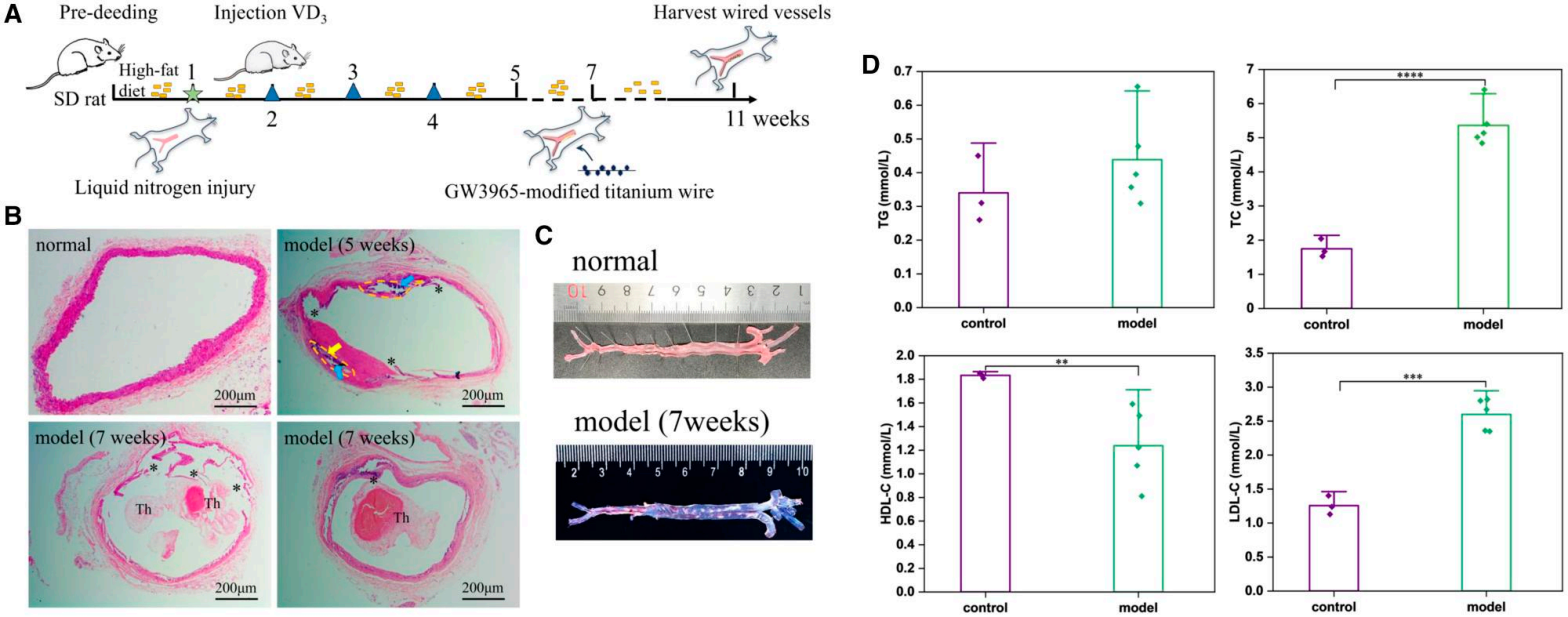
Establishment of rat model of atherosclerosis. (**A**) Summary of the process of modeling and subsequent sample implantation in atherosclerotic rats. (**B**) H&E staining of normal, after injury and high fat feeding for 5- and 7-week rat abdominal aorta. (**C**) Aorta gross oil red staining of two random rats after modeling for 7 weeks and (**D**) blood lipid testing (mean±SD, *N* ≥ 3, *P ˂ 0.05, **P ˂ 0.01, ***P ˂ 0.001, ****P ˂ 0.0001).

To evaluate the therapeutic effect of GW3965-modified materials against AS, atherosclerotic rat models were used. In brief, SD rats were pre-fed with a high-cholesterol diet for 1 week, and the abdominal aorta was treated with frostbite by liquid nitrogen. Subsequently, vitamin D_3_ (VD_3_) was injected intraperitoneally at 2, 3 and 4 weeks to speed up the model process. After 7 weeks, we constructed an atherosclerotic lesion with featured thrombi, and performed an additional 4 weeks of sample implantation, as in Figure 13A. Liquid nitrogen entered the endothelium and was gasified immediately, leading to endothelial injury by cold-induced. When the endothelium was frozen and thawed instantly, the membranous structure was extensively destroyed, and the endothelial cells underwent apoptosis or death due to the formation of ice crystals intra- and extracellular [43]. With the breakdown of the barrier function, AS occurred. VD_3_, as a calcium inducer, promoted the absorption of blood calcium. High-fat diet increased the content of blood lipids in rats, which made lipids easily deposited in the vascular wall, and then combined with calcium deposition to promote vascular calcification, thereby accelerating the formation of arterial lesions and plaques. H&E staining in Figure 13B showed that, compared with normal rat abdominal aorta, there was significant plaque formation 5 weeks after modeling, accompanied by incomplete endodermis. The necrotic core within the plaque (highlighted by the yellow dotted line) contained a large amount of calcification (blue arrow) and foam cells (yellow arrow). Vascular calcification could lead to reduced vascular plasticity and increase the risk of blood vessel rupture and bleeding. At 7 weeks, it was obvious that thrombosis caused by plaque rupture eventually led to blood vessel blockage. The sections of two random models showed that the animal models were histologically characterized by necrotic cores and thrombi. In addition, there was a large accumulation of lipids in the abdominal aorta of the rats at 7 weeks and they were stained with oil red (Figure 13C). The changes of blood components in the model group were tested. According to the four results of blood lipids, TG in the model group had no significant difference compared with the normal group, while TC and LDL-C increased by 67% and 52%, respectively. Meanwhile, HDL-C levels decreased significantly, indicating that the model animals were in a typical state of high cholesterol. To sum up, a rat model of AS with necrotic cores and thrombi has been successfully established, which can be used to investigate the therapeutic benefits of samples *in vivo*.

**Figure 14.**
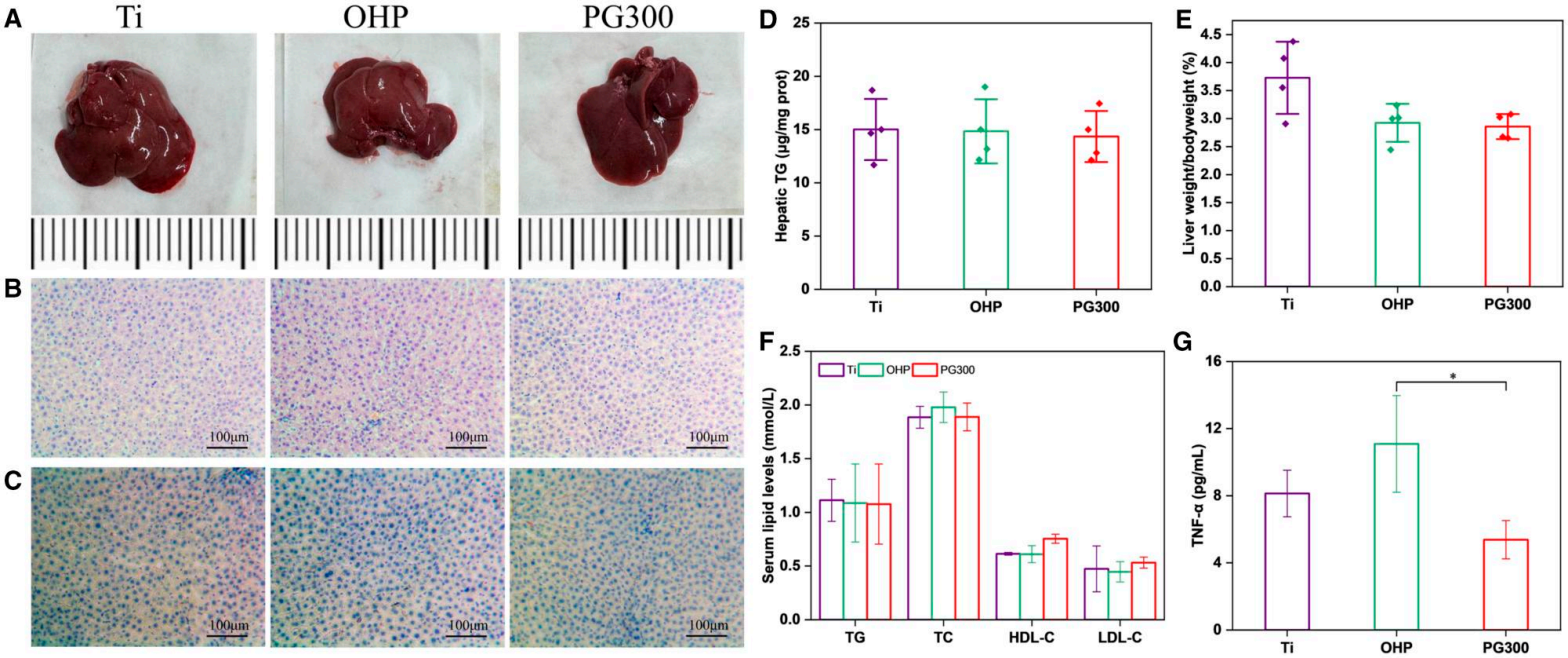
Detection of GW3965-modified material in liver and serum lipid. After treatment, liver and serum samples were collected and analyzed as follows: (**A**) Liver photographs. (**B**) H&E staining and (**C**) Oil red O staining of liver. (**D**) TG quantitative analysis with total liver lipid extract. (**E**) Liver weight/bodyweight ratio, (**F**) serum lipid levels and (**G**) TNF-α levels (mean±SD, *N* ≥ 3, *P ˂ 0.05).

**Figure 15.**
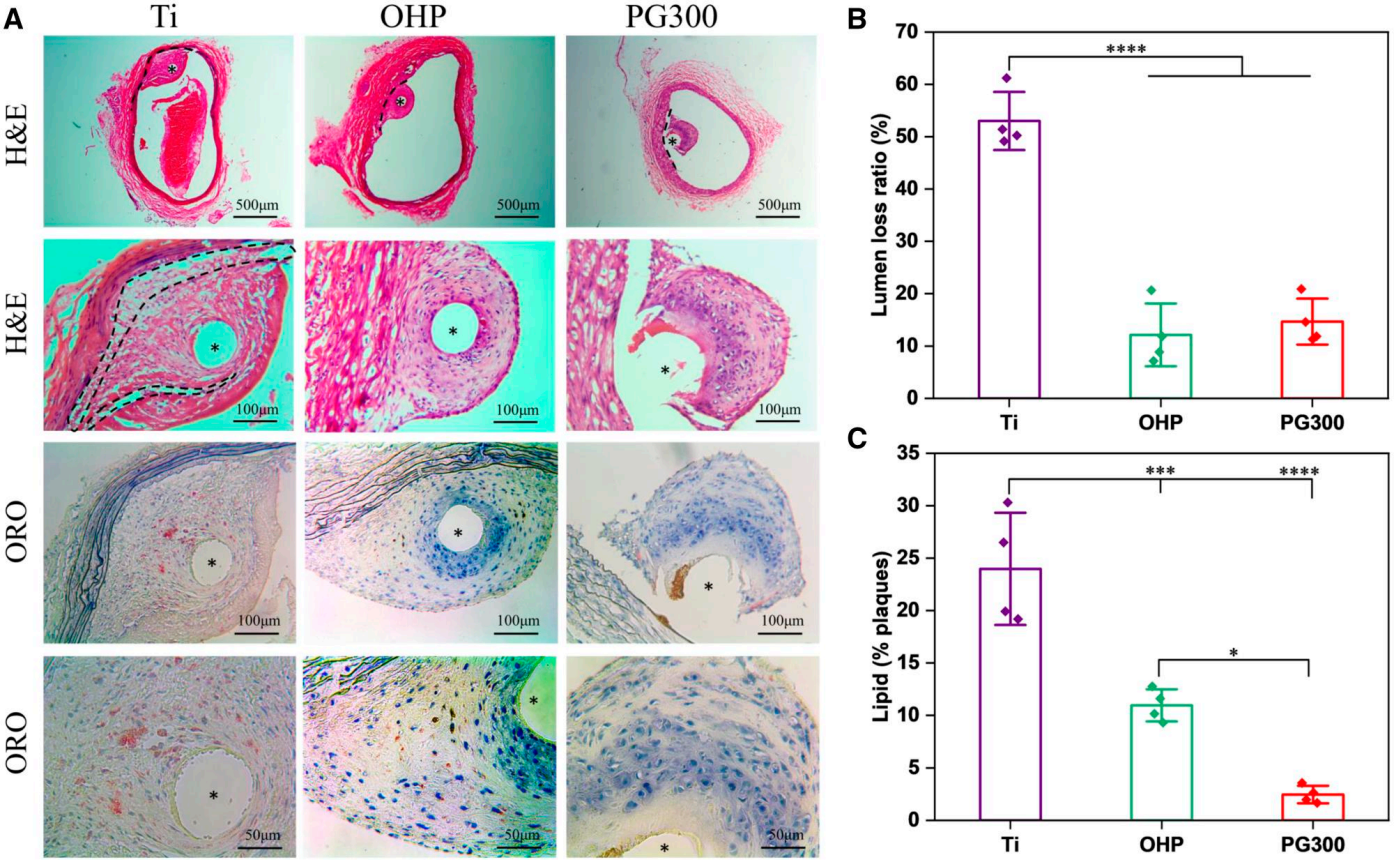
*In vivo* treatment of GW3965-modified material. (**A**) H&E staining and oil red O staining. Quantitative statistics of (**B**) lumen loss ratio and (**C**) lipid within the plaque (mean±SD, *N* ≥ 3, *P ˂ 0.05, ***P ˂ 0.001, ****P ˂ 0.0001).

Since treatment with LXR agonists can cause liver toxicity, we first explored the effects of GW3965-modified materials on the liver. Four weeks after implantation of the samples into the abdominal aorta of the AS model rats, the livers of the rats were taken for digital photographs and weighed to calculate the liver weight to body weight ratio, and the liver triglyceride level was further measured (Figure 14A, D and E). There were no significant differences in liver size, color, weight and triglycerides, indicating that the modified material did not induce lipid accumulation in the liver. The results of H&E staining and oil red O staining further confirmed that the modified material was not hepatotoxic (Figure 14B and C). In addition, the blood of rats was taken for the detection of four items of blood lipids and inflammatory factors, and the results showed that each index was similar, while the inflammatory factors were significantly reduced (Figure 14F and G). In general, the modified material relieved the inflammation without causing an increase in liver and blood triglyceride levels. To verify the *in vivo* therapeutic effect of the material, we dissected the implanted site of the abdominal aorta and performed a staining analysis. Figure 15A showed that the vascular lumen of the Ti had neo-AS and thrombosis, resulting in vascular stenosis. In addition, the endodermis of the Ti and OHP was still in an incomplete state and there were necrotic cores under the subendothelial. In contrast, the intimal tissue of PG300 was relatively complete. The vascular lumen loss ratios of Ti, OHP, and PG300 were 51%, 13% and 14%, respectively (Figure 15B), indicating that PAMAM and GW3965 improved thrombosis and lumen loss ratios in the AS model. The neo-atheromatous plaques of Ti and OHP had a large accumulation of foam cells (dotted line circled area) and were stained red by oil red O. Compared with Ti and OHP, the lipid content of PG300 decreased by 90% and 78%, respectively (Figure 15C), indicating that the GW3965 modified material significantly reduced the lipid accumulation in the plaque and effectively improved the vascular patency rate, this is consistent with previous reports [46, 47].

## Discussion

PCI is a major clinical treatment for cardiovascular diseases however its biocompatibility is inadequate at present, and complications such as late stent thrombosis and restenosis have always existed. Under pathological conditions, the environment of atherosclerotic plaque is complex, involving a variety of cells and signaling pathways, among which macrophages play a significant role in the occurrence and development of AS [48]. In the early stage of AS, an increased number of macrophages leads to the elimination of lipids and inflammation. However, as macrophages phagocytose excess lipoproteins, plaque macrophages become functionally weakened which are reflected in the decreased migration ability, phagocytic efficiency and anti-inflammatory effects, further promoting the development of complex ruptured plaques. These unstable plaques may eventually result in plaque rupture and local arterial thrombosis [12, 49]. Macrophages are pluripotent cells with strong plasticity, involved in lipid metabolism, immunity and efferocytosis; however, the current study of AS macrophages mostly concentrated in the targeted therapy. Therefore, in this work, we introduced a compound coating that regulated macrophage function through multichannel on the material surface to deliver the drug *in situ* to atherosclerotic plaques, providing the foundation for the feasibility of implementing interventional therapy.

**Figure 16.**
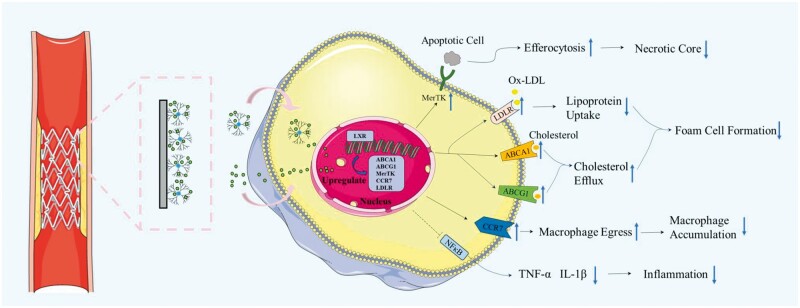
Changes in signaling pathways of macrophages associated with atherosclerosis following treatment with LXR agonist-modified materials.

LXR agonists are promising for the treatment of AS, but when administered systemically at therapeutic doses, they cause accumulation of hepatic lipids and elevation of serum triglyceride levels [38]. In this study, an LXR agonist (GW3965) was introduced on the material surface and delivered *in situ* to the target cells within the plaques to increase drug utilization while avoiding the side effects of systemic administration. Triglyceride in hepatic and serum and other biochemical markers in serum such as cholesterol showed no differences between any group (Figure 14), as well as the ratio of liver weight to body weight. Remarkably, after treatment with LXR agonists, the levels of TNF-α in the serum significantly reduced. Taken together, these results suggested that the GW3965-modified material can be used to treat AS without confounding effects on serum and hepatic lipid levels. AS is a reversible dynamic process, and the number and phenotype of macrophages influence plaque progression and regression. Studies have shown that cholesterol reduction can effectively reduce the number of macrophages and alleviate inflammation, and effective efferocytosis can also affect metabolism and inflammation [50]. Thus, the reduction of plaque macrophage content by promoting macrophage efferocytosis, migration, or polarization toward a pro-involuting M2 phenotype, combined with reduction of cholesterol content and restoration of efferocytosis, is an effective means of atherogenic regression [11]. Combined with *in vivo* and *in vivo* experiments, it was revealed that the ability of GW3965 modified material on macrophages to induce regression of atherosclerotic plaques may be due to the following mechanisms. (i) Regulating lipid metabolism of macrophages in lesions to reduce foam cell formation, which was achieved by inducing the expression of ABCA1 and ABCG1 to increase cholesterol efflux and by downregulating the expression of LDLR to inhibit lipid uptake, (ii) increased expression of MerTK to reduce the amount of apoptotic cells and further reduce the necrotic core; (iii) upregulation of chemokine receptor CCR7, a factor functionally required for regression, to reduce the number of macrophages, and (iv) inhibited the release of inflammatory cytokines to alleviate the persistent inflammation in plaques (Figure 16). Overall, the study established an *in situ* therapeutic approach beyond targeting macrophages for the treatment of AS. In contact with the pathological microenvironment, the molecules released from the materials entered the cells and affected the gene expression. Conjunctively, through regulating AS involved molecules such as oxLDL and inflammatory factor [51], thereby mediating the change of cell behavior to achieve the therapeutic effect.

The number of foam cells in atherosclerotic plaques depends on cell recruitment, *in situ* proliferation, migration, and death. We used oxLDL to stimulate macrophages to mimic foam cells in plaques *in vitro* and found that GW3965-modified material decreased the adhesion of normal macrophages as well as foam cells. Coupled with the upregulation of CCR7 expression, this suggests that GW3965 modified material may affect the number of foam cells from two aspects. For one thing, it worked by regulating the intracellular cholesterol levels, and for another, it was to reverse the migration of cholesterol-loaded cells. Moreover, the reduction of inflammatory macrophages and foam cells also reduced persistent inflammation within the plaque to a certain extent (Figure 5). However, inconsistent with previous reports [52–54], GW3965 modified material did not show induction of macrophage reprogramming, which was a phenotypic transformation of macrophages from M1 into M2. According to the FCM, M1 was significantly decreased, while M2 was only slightly increased with no significant difference, indicating that GW3965-modified material might inhibit inflammation mainly by reducing the number of M1 macrophages. This phenomenon may be due to the difference in the amount of grafting and release on the surface compared with the nanosystem and probably the limited polarization ability of RAW264.7 cell line. Furthermore, we also explored the effects of the material on the intracellular molecular level, and the regulation of GW3965-modified materials on macrophages, apart from staying at the cellular level, changed their inflammatory, oxidative and lipid states at the molecular level.

For biomaterials in implanted medical devices, excellent hemocompatibility is the premise of implantation safety, and thrombosis after implantation is also an urgent problem to be solved. Ti-based materials are often as implant material [55], due to superior biocompatibility, but the high thrombosis activity [56] induced Ti has limited its use clinically, in this work, we also verified the blood compatibility of the modified material on the basis of Ti. After alkali activation treatment on Ti, the increase of roughness and complex microstructure enhanced platelet adhesion, activation and aggregation and release [57]. The introduction of PAMAM presumably smoothed the substrate surface morphology and shifted microstructure toward the less complex, which reduced the platelet adhesion and activation, and promoted the anticoagulant properties. Concurrently, PAMAM improved the hydrophilicity of the material, and the albumin adsorbed on the hydrophilic surface had a blocking effect on the adsorption of platelets for a certain time [58]. Collectively, OHP material appeared good anticoagulant effect in a short time. LXR agonists have been extensively studied in lipid and inflammation, but few have been reported in anticoagulation. Based on the ability of LXR ligands to regulate platelet function by inhibiting calcium release, which is the initial step of platelet activation, Spyridon et al. [59] proposed another mechanism that may contribute to the antiatherogenic effects of LXR ligands scilicet their nongenomic antithrombotic effects. GW3965 modified materials presented high absorbance and BCI within 30 min, while the anticoagulant effect of PG500 decreased over a longer period of time (45–60 min), especially at 60 min. It may be that the material surface or blood cells except platelets in the blood have an effect on coagulation after the longer contact time of the material with whole blood, thus reducing BCI on the surface of PG500 material.

## Conclusion

In this study, we proposed a therapeutic surface engineering strategy to regulate the pathological microenvironment by introducing the GW3965 into the device surface to intervene *in situ* AS regression from the perspective of material biology. In conclusion, our work demonstrated that the GW3965-modified material exhibited anti-atherosclerotic capability, which may be mainly due to its genetic regulation of macrophage functions through multichannel, including lipid metabolism, inflammation, efferocytosis and migration, combined with its non-genetic antithrombotic effects. Therefore, as an implant material for the treatment of AS, it not only reduced the plaque burden of the pre-existing lesions but also reduced the risk of thrombosis. Collectively, the GW3965-modified material is promising in the treatment of AS and can be further explored as a vascular stent.

## Supplementary Material

rbae089_Supplementary_Data
