# Supplementary material for: Therapeutic biomaterials with liver X receptor agonists based on the horizon of material biology to regulate atherosclerotic plaque regression in situ for devices surface engineering
Source: Regen Biomater. 2024 Aug 6;11:rbae089. doi: 10.1093/rb/rbae089 (PMC11335375; doi:10.1093/rb/rbae089)
Supplement: rbae089_Supplementary_Data [file rbae089_supplementary_data.docx]

Table S1. Percentage of atoms on the surface of different samples.
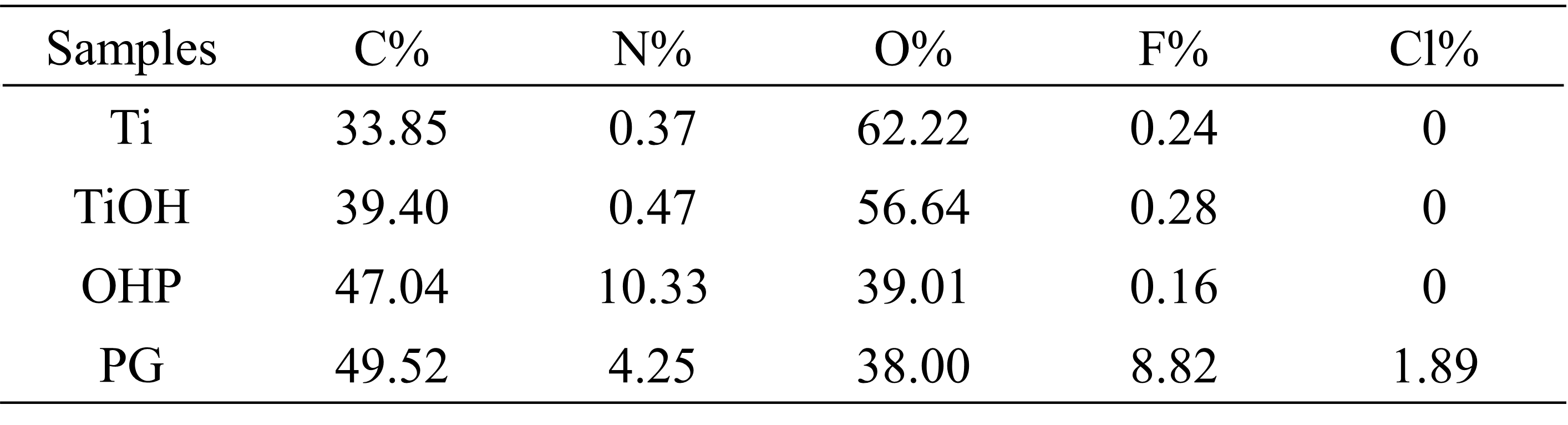


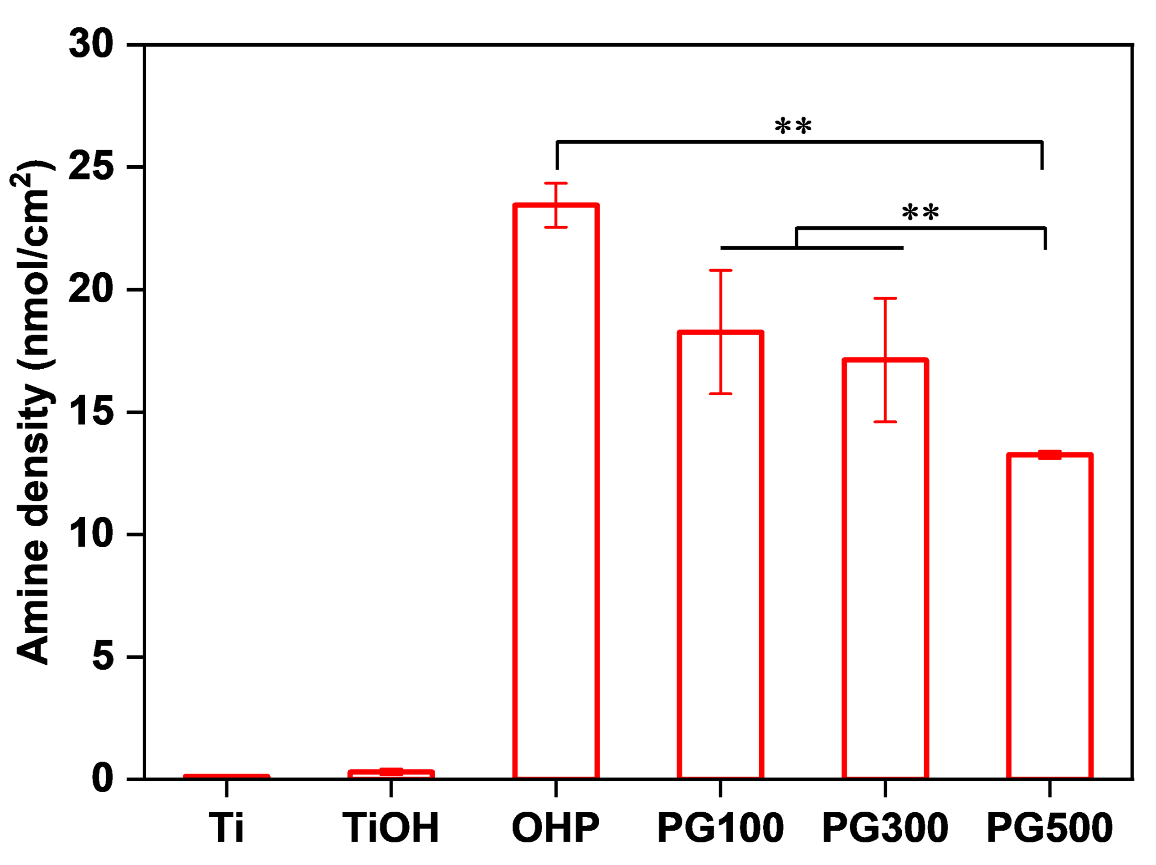


Figure S1. Amine density of different samples after 30 days of release. (mean±SD, N=3, p ˂ 0.05 *, p ˂ 0.01 **, p ˂ 0.001 *** and p ˂ 0.0001****)


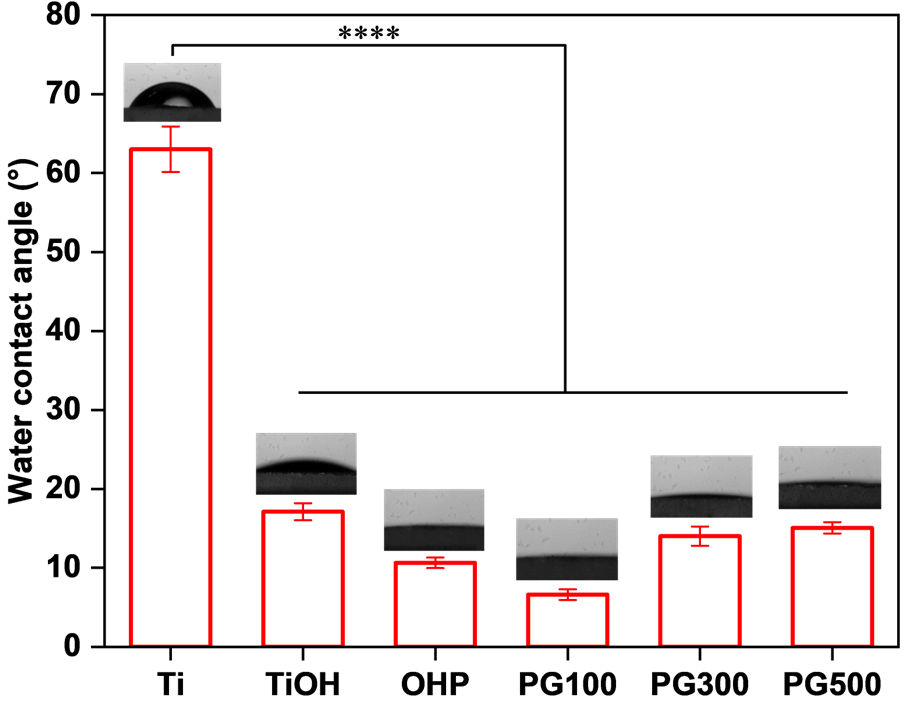


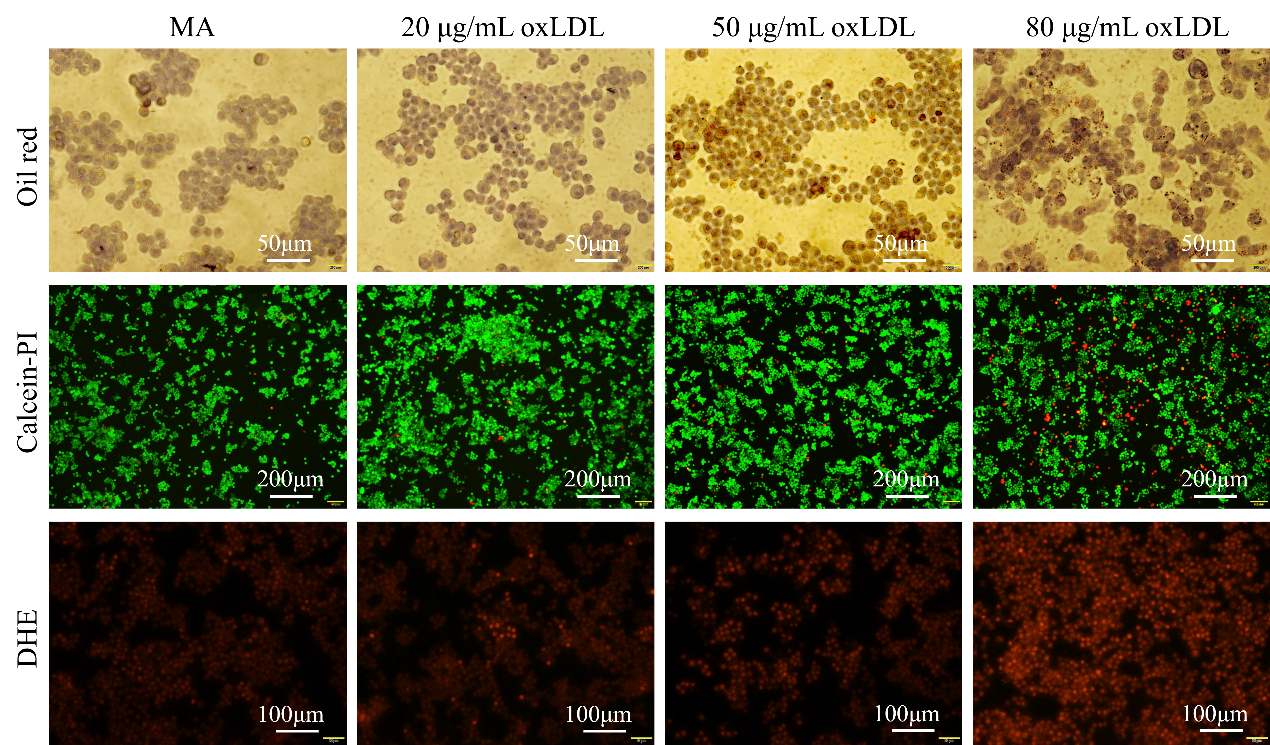
Figure S2. Water contact angle of different samples. (mean±SD, N=3, p ˂ 0.05 *, p ˂ 0.01 **, p ˂ 0.001 *** and p ˂ 0.0001****)

Figure S3. Results of a foam cell model of macrophages stimulated with different concentrations of oxLDL.


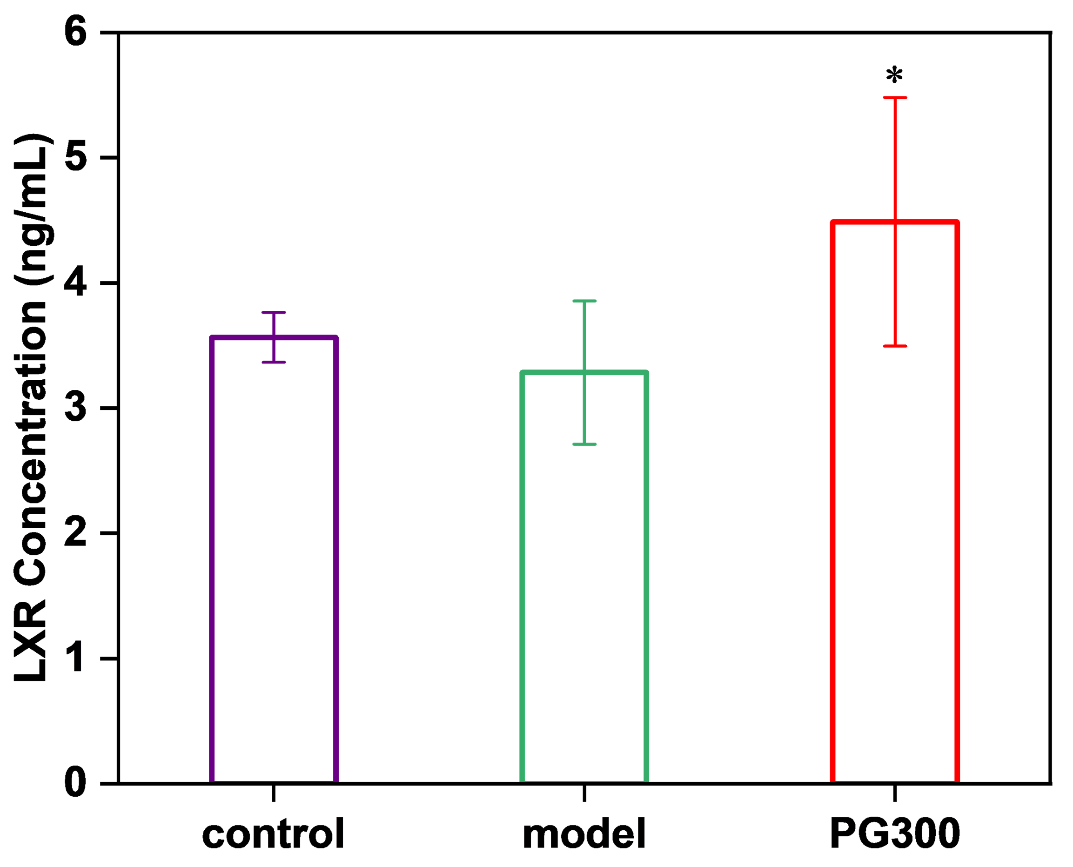


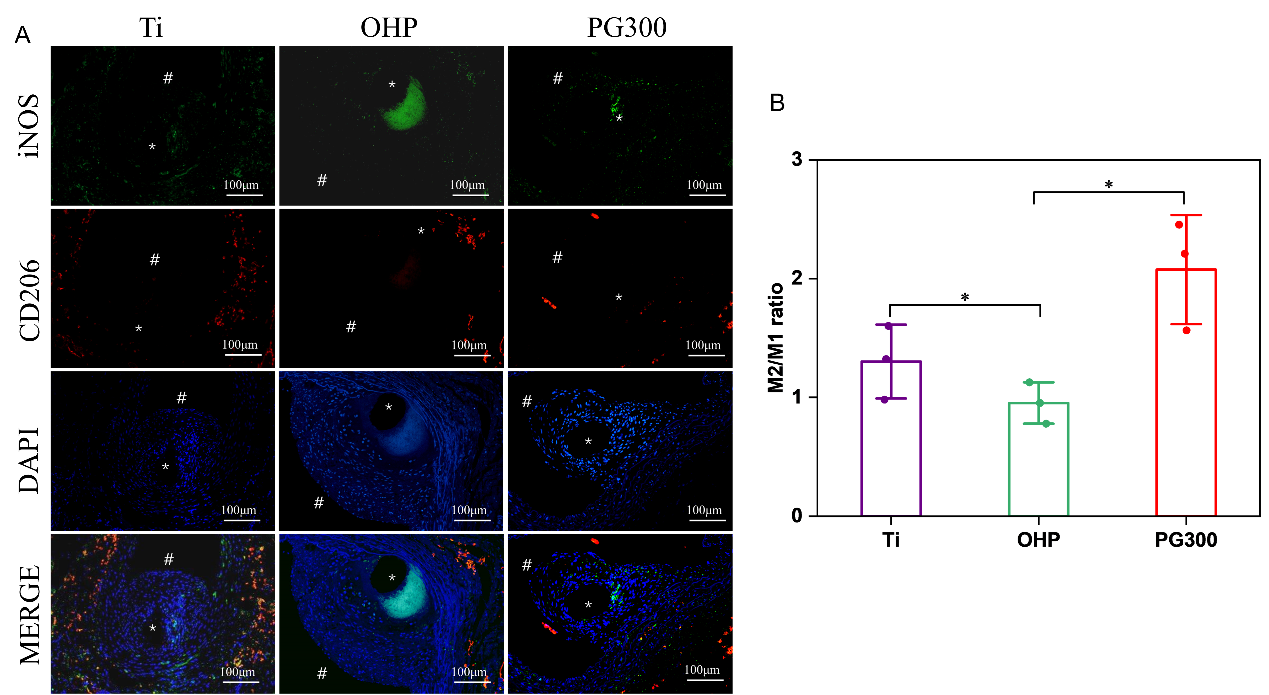
Figure S4. Changes of mRNA expression of LXR in macrophages after PG300 treatment by Elisa kits. (mean±SD, N=3, p ˂ 0.05 *, p ˂ 0.01 **, p ˂ 0.001 *** and p ˂ 0.0001****)

Figure S5. Phenotypic characterization of macrophages in plaque tissue. (A) Immunofluorescence images of M1 macrophages (iNOS+ labeled) and M2 macrophages (CD206+ labeled). (B) The ratio of M2 to M1. (mean±SD, N=3, p ˂ 0.05 *, p ˂ 0.01 **, p ˂ 0.001 *** and p ˂ 0.0001****)
